# Supplementary material for: Sustainability of religious communities
Source: PLoS One. 2021 May 7;16(5):e0250718. doi: 10.1371/journal.pone.0250718 (PMC8104927; doi:10.1371/journal.pone.0250718)
Supplement: S1 Fig — (DOCX) [file pone.0250718.s001.docx]

(01) Active believers=

INTEG (Conversion+Discipleship-Loss belief,1)

Units: person

(02) church members=

Active believers+Passive believers

Units: person

(03) Conversion=

Active believers*conversion rate*conversion fraction

Units: person/Year

(04) conversion duration=

5

Units: Year

(05) conversion fraction=

0.5

Units: Dmnl

(06) conversion rate=

probability of contacting unbelievers *sustainable potential / conversion duration

Units: Dmnl

(07) disciple rate=

0.25

Units: Dmnl

(08) Discipleship = A FUNCTION OF( disciple rate,Passive believers)

Discipleship=

Passive believers * disciple rate / disciple duration

Units: person/Year

(09) Evangelism=

Unbelievers*(Passive believers/Total population)*evangelism fraction*sustainable potential

/evangelism duration

Units: person/Year

(10) evangelism duration=

2

Units: Year

(11) evangelism fraction=

0.5

Units: Dmnl

(12) FINAL TIME = 30

Units: Year

The final time for the simulation.

(13) INITIAL TIME = 0

Units: Year

The initial time for the simulation.

(14) Loss belief=

Active believers*loss rate

Units: person/Year

(15) loss rate=

0.01

Units: Dmnl

(16) Passive believers= INTEG (Evangelism-Discipleship,50)

Units: person

(17) probability of contacting unbelievers=

Unbelievers/Total population

Units: **undefined**

(18) SAVEPER =

TIME STEP

Units: Year [0,?]

The frequency with which output is stored.

(19) sustainable potential=

4

Units: 1/person

(20) TIME STEP = 0.125

Units: Year [0,?]

The time step for the simulation.

(21) Total population=

Active believers+Passive believers+Unbelievers

Units: person

(22) Unbelievers= INTEG (

Loss belief-Conversion-Evangelism,950)

Units: person
