# Supplementary material for: Sustainability of religious communities
Source: PLoS One. 2021 May 7;16(5):e0250718. doi: 10.1371/journal.pone.0250718 (PMC8104927; doi:10.1371/journal.pone.0250718)
Supplement: S2 Fig — (DOCX) [file pone.0250718.s002.docx]

Time PBs1 PBs2 PBs3 CMs1 CMs2 CMs3

0.125 49.7396 50.7292 51.7188 50.9896 51.9792 52.9688

0.25 49.4795 51.4648 53.487 51.993 54.0277 56.0993

0.375 49.2197 52.2067 55.3052 53.0105 56.1476 59.399

0.5 48.9603 52.9546 57.1738 54.0421 58.341 62.8755

0.625 48.7012 53.7085 59.0932 55.0881 60.6098 66.5368

0.75 48.4424 54.468 61.0634 56.1485 62.9563 70.3907

0.875 48.1838 55.2329 63.0847 57.2237 65.3825 74.4455

1 47.9256 56.003 65.1568 58.3138 67.8906 78.7094

1.125 47.6677 56.778 67.2797 59.4188 70.4827 83.1907

1.25 47.4101 57.5575 69.4529 60.5391 73.1611 87.8979

1.375 47.1528 58.3414 71.6758 61.6748 75.928 92.8394

1.5 46.8957 59.1291 73.9477 62.8259 78.7855 98.0238

1.625 46.6389 59.9205 76.2677 63.9928 81.7359 103.46

1.75 46.3824 60.7151 78.6346 65.1756 84.7813 109.155

1.875 46.1262 61.5126 81.0469 66.3745 87.9242 115.118

2 45.8702 62.3124 83.5031 67.5896 91.1665 121.357

2.125 45.6145 63.1143 86.0011 68.8211 94.5106 127.879

2.25 45.359 63.9176 88.5388 70.0691 97.9586 134.692

2.375 45.1038 64.722 91.1136 71.334 101.513 141.804

2.5 44.8488 65.527 93.7229 72.6157 105.175 149.22

2.625 44.5941 66.3319 96.3634 73.9146 108.948 156.946

2.75 44.3396 67.1364 99.0318 75.2307 112.833 164.988

2.875 44.0854 67.9397 101.724 76.5642 116.833 173.352

3 43.8314 68.7413 104.437 77.9154 120.948 182.039

3.125 43.5776 69.5405 107.165 79.2843 125.183 191.055

3.25 43.3241 70.3368 109.904 80.6712 129.537 200.4

3.375 43.0707 71.1295 112.649 82.0763 134.013 210.075

3.5 42.8176 71.9178 115.394 83.4996 138.613 220.081

3.625 42.5647 72.701 118.134 84.9413 143.338 230.417

3.75 42.3121 73.4785 120.863 86.4017 148.189 241.078

3.875 42.0596 74.2495 123.574 87.8809 153.169 252.062

4 41.8073 75.0131 126.261 89.379 158.277 263.364

4.125 41.5553 75.7686 128.918 90.8963 163.516 274.975

4.25 41.3035 76.5152 131.537 92.4328 168.886 286.887

4.375 41.0518 77.252 134.111 93.9887 174.388 299.091

4.5 40.8004 77.9782 136.633 95.5643 180.023 311.575

4.625 40.5491 78.693 139.096 97.1596 185.791 324.324

4.75 40.2981 79.3953 141.493 98.7748 191.692 337.326

4.875 40.0473 80.0844 143.816 100.41 197.727 350.563

5 39.7966 80.7594 146.058 102.065 203.896 364.017

5.125 39.5462 81.4193 148.212 103.741 210.197 377.669

5.25 39.296 82.0632 150.271 105.438 216.632 391.498

5.375 39.0459 82.6902 152.23 107.155 223.198 405.482

5.5 38.796 83.2993 154.08 108.892 229.895 419.599

5.625 38.5464 83.8897 155.818 110.651 236.722 433.825

5.75 38.2969 84.4604 157.436 112.431 243.678 448.134

5.875 38.0476 85.0106 158.93 114.232 250.76 462.5

6 37.7986 85.5392 160.296 116.054 257.967 476.899

6.125 37.5497 86.0454 161.529 117.898 265.296 491.303

6.25 37.301 86.5284 162.627 119.763 272.745 505.687

6.375 37.0525 86.9872 163.585 121.65 280.312 520.024

6.5 36.8042 87.421 164.403 123.559 287.992 534.287

6.625 36.5561 87.829 165.078 125.49 295.784 548.451

6.75 36.3082 88.2104 165.61 127.443 303.683 562.492

6.875 36.0605 88.5644 165.998 129.417 311.686 576.385

7 35.8131 88.8903 166.243 131.415 319.788 590.106

7.125 35.5658 89.1873 166.346 133.434 327.986 603.634

7.25 35.3187 89.4549 166.308 135.476 336.274 616.948

7.375 35.0719 89.6925 166.132 137.541 344.649 630.028

7.5 34.8253 89.8993 165.82 139.628 353.104 642.857

7.625 34.5789 90.075 165.376 141.737 361.634 655.418

7.75 34.3327 90.219 164.803 143.87 370.235 667.695

7.875 34.0867 90.3309 164.106 146.025 378.9 679.677

8 33.841 90.4103 163.289 148.203 387.623 691.351

8.125 33.5955 90.4569 162.357 150.404 396.399 702.707

8.25 33.3502 90.4705 161.315 152.629 405.221 713.738

8.375 33.1052 90.4508 160.168 154.876 414.083 724.436

8.5 32.8605 90.3977 158.922 157.146 422.979 734.797

8.625 32.616 90.3112 157.583 159.439 431.901 744.816

8.75 32.3717 90.1911 156.157 161.756 440.844 754.493

8.875 32.1278 90.0377 154.649 164.095 449.799 763.827

9 31.8841 89.8508 153.066 166.458 458.762 772.817

9.125 31.6407 89.6308 151.412 168.844 467.724 781.466

9.25 31.3975 89.3779 149.695 171.253 476.68 789.777

9.375 31.1547 89.0923 147.92 173.686 485.621 797.753

9.5 30.9121 88.7745 146.091 176.141 494.543 805.401

9.625 30.6699 88.4248 144.216 178.62 503.436 812.724

9.75 30.428 88.0437 142.298 181.121 512.296 819.73

9.875 30.1864 87.6317 140.344 183.646 521.116 826.425

10 29.9451 87.1895 138.358 186.194 529.888 832.818

10.125 29.7042 86.7176 136.345 188.765 538.607 838.916

10.25 29.4636 86.2168 134.309 191.359 547.267 844.727

10.375 29.2234 85.6878 132.255 193.975 555.862 850.26

10.5 28.9835 85.1313 130.186 196.615 564.386 855.525

10.625 28.744 84.5482 128.107 199.277 572.832 860.53

10.75 28.5049 83.9393 126.021 201.962 581.197 865.285

10.875 28.2662 83.3056 123.932 204.669 589.474 869.798

11 28.0279 82.6479 121.842 207.399 597.659 874.079

11.125 27.79 81.9673 119.755 210.151 605.747 878.137

11.25 27.5526 81.2646 117.673 212.925 613.734 881.982

11.375 27.3155 80.5409 115.599 215.721 621.615 885.621

11.5 27.079 79.7971 113.535 218.54 629.386 889.065

11.625 26.8429 79.0345 111.484 221.38 637.044 892.321

11.75 26.6072 78.2539 109.447 224.241 644.586 895.398

11.875 26.372 77.4564 107.427 227.124 652.008 898.304

12 26.1374 76.6431 105.424 230.028 659.307 901.048

12.125 25.9032 75.815 103.44 232.954 666.481 903.637

12.25 25.6696 74.9732 101.477 235.9 673.528 906.079

12.375 25.4365 74.1187 99.536 238.867 680.445 908.38

12.5 25.2039 73.2526 97.6175 241.854 687.232 910.549

12.625 24.9719 72.3759 95.7228 244.862 693.885 912.591

12.75 24.7404 71.4896 93.8527 247.889 700.405 914.513

12.875 24.5096 70.5948 92.0078 250.936 706.79 916.322

13 24.2793 69.6924 90.1888 254.003 713.039 918.023

13.125 24.0497 68.7834 88.3962 257.09 719.152 919.622

13.25 23.8207 67.8687 86.6304 260.195 725.129 921.125

13.375 23.5923 66.9493 84.8917 263.319 730.969 922.536

13.5 23.3646 66.026 83.1804 266.461 736.673 923.861

13.625 23.1375 65.0998 81.4967 269.621 742.24 925.105

13.75 22.9111 64.1715 79.8408 272.8 747.673 926.272

13.875 22.6855 63.2419 78.2126 275.995 752.97 927.366

14 22.4605 62.3118 76.6124 279.208 758.134 928.392

14.125 22.2363 61.382 75.04 282.438 763.165 929.352

14.25 22.0128 60.4531 73.4953 285.685 768.064 930.252

14.375 21.79 59.526 71.9783 288.948 772.833 931.095

14.5 21.5681 58.6013 70.4888 292.226 777.474 931.883

14.625 21.3469 57.6796 69.0267 295.52 781.987 932.62

14.75 21.1265 56.7616 67.5917 298.829 786.374 933.309

14.875 20.907 55.8478 66.1836 302.153 790.637 933.953

15 20.6883 54.9388 64.8023 305.491 794.779 934.554

15.125 20.4704 54.0351 63.4473 308.843 798.801 935.115

15.25 20.2534 53.1372 62.1184 312.209 802.704 935.638

15.375 20.0373 52.2456 60.8153 315.588 806.492 936.126

15.5 19.822 51.3607 59.5377 318.979 810.166 936.581

15.625 19.6077 50.4829 58.2852 322.383 813.729 937.004

15.75 19.3943 49.6127 57.0576 325.799 817.182 937.398

15.875 19.1819 48.7503 55.8544 329.226 820.529 937.765

16 18.9704 47.8961 54.6753 332.664 823.771 938.105

16.125 18.7599 47.0504 53.5199 336.113 826.91 938.421

16.25 18.5503 46.2134 52.3879 339.571 829.949 938.715

16.375 18.3418 45.3855 51.2789 343.04 832.891 938.987

16.5 18.1343 44.5669 50.1924 346.517 835.737 939.238

16.625 17.9278 43.7578 49.1282 350.003 838.49 939.471

16.75 17.7224 42.9583 48.0859 353.498 841.152 939.686

16.875 17.5181 42.1687 47.065 357 843.727 939.885

17 17.3148 41.389 46.0652 360.509 846.215 940.068

17.125 17.1126 40.6195 45.0861 364.025 848.619 940.237

17.25 16.9115 39.8602 44.1274 367.547 850.941 940.392

17.375 16.7115 39.1113 43.1886 371.075 853.185 940.535

17.5 16.5127 38.3728 42.2694 374.608 855.351 940.665

17.625 16.315 37.6447 41.3694 378.146 857.443 940.785

17.75 16.1185 36.9272 40.4883 381.688 859.462 940.894

17.875 15.9232 36.2203 39.6256 385.234 861.41 940.993

18 15.7291 35.5239 38.7811 388.783 863.29 941.083

18.125 15.5361 34.8382 37.9544 392.335 865.103 941.165

18.25 15.3444 34.163 37.1451 395.889 866.852 941.239

18.375 15.1539 33.4985 36.3529 399.444 868.539 941.305

18.5 14.9647 32.8445 35.5774 403.001 870.165 941.365

18.625 14.7767 32.2011 34.8184 406.558 871.732 941.418

18.75 14.5899 31.5681 34.0754 410.115 873.243 941.465

18.875 14.4045 30.9457 33.3482 413.672 874.699 941.506

19 14.2203 30.3336 32.6364 417.228 876.101 941.543

19.125 14.0375 29.7318 31.9397 420.782 877.452 941.574

19.25 13.8559 29.1404 31.2579 424.334 878.754 941.602

19.375 13.6757 28.5591 30.5905 427.884 880.007 941.625

19.5 13.4968 27.9879 29.9373 431.431 881.214 941.644

19.625 13.3193 27.4267 29.2981 434.974 882.376 941.659

19.75 13.1431 26.8755 28.6725 438.513 883.494 941.671

19.875 12.9682 26.334 28.0602 442.047 884.571 941.68

20 12.7948 25.8023 27.461 445.577 885.607 941.687

20.125 12.6227 25.2803 26.8745 449.1 886.604 941.69

20.25 12.452 24.7677 26.3006 452.618 887.563 941.692

20.375 12.2827 24.2645 25.7389 456.129 888.485 941.691

20.5 12.1148 23.7707 25.1893 459.633 889.373 941.688

20.625 11.9483 23.286 24.6513 463.129 890.226 941.683

20.75 11.7832 22.8103 24.1249 466.617 891.047 941.676

20.875 11.6196 22.3436 23.6097 470.096 891.836 941.668

21 11.4574 21.8857 23.1056 473.567 892.595 941.658

21.125 11.2966 21.4365 22.6122 477.028 893.325 941.648

21.25 11.1373 20.9959 22.1293 480.479 894.026 941.635

21.375 10.9794 20.5637 21.6568 483.919 894.699 941.622

21.5 10.8229 20.1398 21.1944 487.348 895.347 941.608

21.625 10.668 19.7242 20.7419 490.766 895.969 941.593

21.75 10.5144 19.3166 20.2991 494.173 896.567 941.577

21.875 10.3624 18.9169 19.8657 497.566 897.141 941.56

22 10.2118 18.525 19.4416 500.948 897.693 941.543

22.125 10.0627 18.1409 19.0266 504.316 898.222 941.525

22.25 9.91501 17.7643 18.6205 507.67 898.731 941.506

22.375 9.76883 17.3951 18.2231 511.011 899.22 941.488

22.5 9.62413 17.0333 17.8341 514.337 899.689 941.468

22.625 9.4809 16.6787 17.4535 517.648 900.139 941.449

22.75 9.33916 16.3311 17.081 520.944 900.572 941.429

22.875 9.19888 15.9905 16.7165 524.225 900.987 941.409

23 9.06009 15.6567 16.3598 527.49 901.385 941.389

23.125 8.92278 15.3296 16.0108 530.738 901.767 941.368

23.25 8.78694 15.0091 15.6692 533.97 902.134 941.348

23.375 8.65258 14.6951 15.3349 537.185 902.486 941.327

23.5 8.51969 14.3874 15.0078 540.383 902.824 941.307

23.625 8.38828 14.086 14.6876 543.563 903.148 941.286

23.75 8.25834 13.7907 14.3743 546.724 903.458 941.266

23.875 8.12986 13.5014 14.0677 549.868 903.756 941.245

24 8.00286 13.218 13.7677 552.993 904.042 941.224

24.125 7.87731 12.9404 13.4741 556.099 904.316 941.204

24.25 7.75323 12.6685 13.1867 559.186 904.579 941.184

24.375 7.6306 12.4021 12.9055 562.253 904.831 941.164

24.5 7.50942 12.1413 12.6304 565.301 905.072 941.143

24.625 7.38969 11.8857 12.3611 568.329 905.303 941.124

24.75 7.27141 11.6355 12.0975 571.337 905.525 941.104

24.875 7.15456 11.3904 11.8396 574.324 905.737 941.084

25 7.03915 11.1504 11.5872 577.29 905.941 941.065

25.125 6.92516 10.9153 11.3402 580.236 906.136 941.046

25.25 6.81259 10.6851 11.0985 583.16 906.323 941.027

25.375 6.70144 10.4597 10.8619 586.063 906.502 941.008

25.5 6.59169 10.239 10.6304 588.944 906.673 940.989

25.625 6.48335 10.0228 10.4039 591.804 906.837 940.971

25.75 6.3764 9.81115 10.1822 594.642 906.994 940.953

25.875 6.27084 9.60389 9.9652 597.457 907.145 940.935

26 6.16666 9.40095 9.75286 600.251 907.289 940.917

26.125 6.06385 9.20224 9.54505 603.022 907.426 940.9

26.25 5.9624 9.00768 9.34168 605.771 907.558 940.883

26.375 5.86231 8.81719 9.14265 608.497 907.684 940.866

26.5 5.76357 8.63067 8.94788 611.2 907.805 940.849

26.625 5.66616 8.44806 8.75726 613.88 907.92 940.833

26.75 5.57009 8.26927 8.57071 616.537 908.031 940.817

26.875 5.47533 8.09423 8.38815 619.172 908.136 940.801

27 5.38189 7.92285 8.20948 621.783 908.237 940.785

27.125 5.28975 7.75508 8.03462 624.371 908.333 940.77

27.25 5.1989 7.59082 7.8635 626.935 908.425 940.755

27.375 5.10934 7.43001 7.69603 629.476 908.513 940.74

27.5 5.02104 7.27258 7.53213 631.994 908.598 940.725

27.625 4.93401 7.11847 7.37173 634.488 908.678 940.711

27.75 4.84823 6.96759 7.21476 636.959 908.755 940.697

27.875 4.7637 6.81989 7.06113 639.406 908.828 940.683

28 4.68039 6.6753 6.91078 641.83 908.898 940.669

28.125 4.59831 6.53376 6.76364 644.23 908.965 940.655

28.25 4.51743 6.3952 6.61963 646.606 909.028 940.642

28.375 4.43775 6.25956 6.4787 648.959 909.089 940.629

28.5 4.35927 6.12678 6.34077 651.288 909.147 940.617

28.625 4.28195 5.9968 6.20579 653.593 909.202 940.604

28.75 4.20581 5.86957 6.07368 655.875 909.255 940.592

28.875 4.13081 5.74502 5.94439 658.134 909.305 940.58

29 4.05696 5.62311 5.81785 660.368 909.353 940.568

29.125 3.98425 5.50377 5.69402 662.58 909.399 940.556

29.25 3.91265 5.38695 5.57282 664.768 909.442 940.545

29.375 3.84216 5.2726 5.45421 666.932 909.483 940.534

29.5 3.77276 5.16067 5.33813 669.073 909.523 940.523

29.625 3.70445 5.05111 5.22452 671.191 909.56 940.512

29.75 3.63722 4.94387 5.11333 673.286 909.596 940.502

29.875 3.57105 4.83889 5.00451 675.358 909.63 940.491

30 3.50592 4.73614 4.89801 677.406 909.662 940.481
