# Supplementary material for: Sustainability of religious communities
Source: PLoS One. 2021 May 7;16(5):e0250718. doi: 10.1371/journal.pone.0250718 (PMC8104927; doi:10.1371/journal.pone.0250718)
Supplement: S3 Fig — (DOCX) [file pone.0250718.s003.docx]

Time PBs1 PBs2 PBs3 CMs1 CMs2 CMs3

0 50 50 50 50 50 50

0.02 50 51 51 50 50 50

0.04 50 52 53 50 51 51

0.06 50 53 54 50 52 53

0.08 51 54 55 50 53 54

0.1 51 54 57 51 54 55

0.12 52 55 59 51 54 57

0.14 52 56 61 52 55 59

0.16 52 59 64 52 56 61

0.18 52 60 64 52 59 64

0.2 52 62 65 52 60 64

0.22 51 61 67 52 62 65

0.24 50 61 67 52 62 67

0.26 50 61 68 52 62 67

0.28 50 61 69 52 62 69

0.3 50 61 69 52 62 70

0.32 50 63 70 52 62 70

0.34 52 63 71 52 64 71

0.36 52 63 71 54 65 72

0.38 52 63 72 54 65 72

0.4 52 63 72 54 65 73

0.42 52 64 72 54 65 73

0.44 51 65 72 54 66 73

0.46 51 67 74 54 67 74

0.48 51 67 74 54 69 76

0.5 50 68 74 54 69 76

0.52 49 69 74 55 70 76

0.54 49 69 74 55 71 76

0.56 49 68 74 55 71 76

0.58 49 67 74 56 71 76

0.6 49 69 74 56 71 76

0.62 47 71 74 56 73 76

0.64 47 72 74 56 75 76

0.66 47 73 74 56 76 76

0.68 47 73 74 56 77 76

0.7 45 73 75 56 77 76

0.72 45 73 76 56 77 77

0.74 45 73 77 56 77 78

0.76 45 73 78 56 77 79

0.78 44 73 77 56 77 80

0.8 44 73 77 56 77 80

0.82 44 73 77 56 77 80

0.84 44 73 77 56 77 80

0.86 44 73 76 56 77 80

0.88 44 73 75 56 77 80

0.9 44 73 75 56 77 80

0.92 44 73 75 56 77 80

0.94 44 73 75 56 77 80

0.96 44 73 75 56 77 80

0.98 44 72 76 56 77 80

1 44 72 76 56 77 81

1.02 44 71 76 56 77 81

1.04 44 71 76 56 77 81

1.06 43 70 76 56 77 81

1.08 43 70 76 56 77 82

1.1 43 70 78 56 77 82

1.12 42 70 77 56 78 84

1.14 42 69 77 56 78 84

1.16 42 70 79 56 78 84

1.18 42 70 81 57 79 86

1.2 42 70 83 57 79 88

1.22 42 66 83 57 80 90

1.24 42 65 83 57 80 90

1.26 41 65 83 57 80 90

1.28 41 66 85 57 80 91

1.3 41 68 87 57 82 93

1.32 41 68 87 57 84 96

1.34 41 68 89 57 84 97

1.36 41 68 89 57 84 99

1.38 41 68 90 57 84 100

1.4 42 68 91 57 84 101

1.42 41 69 92 58 84 102

1.44 41 69 92 58 85 104

1.46 41 69 93 58 85 105

1.48 41 69 93 58 85 106

1.5 41 69 93 58 85 106

1.52 42 69 94 58 86 107

1.54 42 69 94 59 86 108

1.56 43 69 94 59 87 109

1.58 43 69 94 60 87 110

1.6 43 70 94 60 87 111

1.62 43 70 95 60 88 111

1.64 43 70 96 60 88 112

1.66 43 70 95 60 88 113

1.68 43 71 93 61 88 113

1.7 43 70 93 61 89 113

1.72 42 73 94 61 89 114

1.74 42 73 94 61 92 115

1.76 42 74 96 61 92 115

1.78 44 76 97 61 93 117

1.8 44 77 97 63 95 118

1.82 44 78 98 63 96 118

1.84 44 78 98 63 97 119

1.86 43 79 97 63 97 119

1.88 44 79 96 63 98 119

1.9 44 79 97 65 98 119

1.92 45 80 97 65 98 121

1.94 45 81 97 66 99 121

1.96 45 82 97 66 101 121

1.98 45 81 97 66 102 121

2 45 81 98 66 102 121

2.02 45 81 100 66 102 122

2.04 46 81 100 67 102 124

2.06 46 81 100 68 103 124

2.08 46 81 100 68 103 124

2.1 46 81 102 68 103 124

2.12 45 81 103 68 103 126

2.14 44 80 103 69 104 127

2.16 43 80 103 69 104 127

2.18 43 80 102 69 104 127

2.2 44 81 102 69 104 127

2.22 45 81 103 70 105 127

2.24 46 81 104 71 105 128

2.26 46 82 105 73 105 129

2.28 45 82 106 73 106 130

2.3 45 82 106 73 106 132

2.32 45 83 107 73 107 132

2.34 45 87 108 73 108 133

2.36 45 87 108 73 112 134

2.38 45 87 112 73 112 135

2.4 44 87 112 73 112 139

2.42 43 87 114 73 112 140

2.44 42 87 114 73 112 142

2.46 42 88 116 73 112 142

2.48 42 88 118 73 113 144

2.5 42 88 118 73 113 147

2.52 42 87 119 73 113 147

2.54 42 87 120 73 113 148

2.56 42 87 119 73 113 149

2.58 41 87 120 73 114 149

2.6 40 87 121 73 114 150

2.62 40 87 121 73 114 151

2.64 40 88 121 73 114 151

2.66 39 88 121 73 115 151

2.68 39 87 122 73 115 151

2.7 39 87 122 73 116 152

2.72 39 87 125 73 116 152

2.74 39 86 128 73 116 155

2.76 38 85 128 73 116 158

2.78 38 84 129 73 116 159

2.8 38 83 129 73 116 160

2.82 38 84 128 73 116 160

2.84 38 84 128 73 117 160

2.86 38 84 128 73 118 160

2.88 38 85 128 73 118 160

2.9 38 85 128 73 119 160

2.92 38 86 129 73 120 161

2.94 39 86 129 73 121 162

2.96 39 86 131 74 121 162

2.98 39 86 133 74 121 164

3 38 85 134 74 121 166

3.02 39 85 136 74 121 167

3.04 39 86 137 75 121 169

3.06 39 87 139 75 123 170

3.08 39 87 139 75 124 172

3.1 40 87 140 75 124 173

3.12 40 86 140 76 124 174

3.14 39 88 140 76 124 174

3.16 38 88 141 76 126 174

3.18 38 88 140 76 126 175

3.2 38 87 141 76 126 177

3.22 37 89 141 76 126 178

3.24 37 89 142 76 128 178

3.26 37 89 144 76 128 179

3.28 37 89 144 76 128 181

3.3 37 89 144 76 129 181

3.32 37 90 145 76 129 181

3.34 37 91 146 76 130 182

3.36 37 95 145 76 131 183

3.38 37 95 146 76 135 184

3.4 37 95 148 76 135 185

3.42 38 95 149 76 135 187

3.44 38 94 151 77 136 188

3.46 37 94 152 77 136 190

3.48 36 94 152 77 136 191

3.5 37 96 153 77 136 191

3.52 37 97 153 78 138 192

3.54 37 97 153 78 139 192

3.56 37 98 153 78 139 194

3.58 37 97 152 78 140 194

3.6 37 96 151 78 140 194

3.62 38 97 152 78 140 194

3.64 38 97 152 79 141 195

3.66 36 98 152 79 141 195

3.68 35 98 151 79 142 196

3.7 35 98 153 79 142 196

3.72 35 98 152 79 142 198

3.74 35 97 153 79 142 198

3.76 35 101 154 79 142 199

3.78 35 101 156 79 146 200

3.8 35 101 159 79 146 202

3.82 35 101 161 79 146 205

3.84 35 103 160 79 147 207

3.86 35 104 162 79 149 207

3.88 35 104 162 79 150 209

3.9 35 104 165 79 150 209

3.92 36 104 166 79 150 212

3.94 35 105 168 80 150 213

3.96 35 107 167 80 151 215

3.98 35 106 167 81 153 215

4 35 106 168 81 154 215

4.02 34 106 169 81 154 216

4.04 35 106 171 81 155 217

4.06 35 106 173 82 155 219

4.08 35 107 172 82 155 221

4.1 35 107 172 82 156 222

4.12 35 109 173 82 156 222

4.14 35 110 175 82 158 223

4.16 35 111 177 82 159 225

4.18 34 112 177 82 160 228

4.2 34 112 180 82 161 228

4.22 34 112 182 82 161 231

4.24 34 112 181 82 162 233

4.26 34 110 181 82 164 235

4.28 34 109 182 82 164 236

4.3 32 110 182 82 164 237

4.32 32 108 181 82 165 238

4.34 32 108 181 82 165 239

4.36 33 108 185 82 167 239

4.38 32 107 188 83 167 243

4.4 32 107 188 83 167 246

4.42 32 107 186 83 167 247

4.44 32 108 189 83 167 247

4.46 32 109 189 83 168 250

4.48 32 109 190 83 169 250

4.5 32 109 192 83 169 252

4.52 32 107 193 83 169 254

4.54 32 108 195 83 169 255

4.56 32 108 197 83 170 259

4.58 32 108 198 83 170 261

4.6 32 111 199 83 170 262

4.62 33 111 201 83 173 263

4.64 33 110 201 84 173 265

4.66 33 109 200 84 173 266

4.68 33 111 200 84 174 266

4.7 32 110 200 84 177 266

4.72 31 111 202 84 177 266

4.74 31 110 203 84 178 268

4.76 32 111 204 84 178 270

4.78 33 111 203 85 179 271

4.8 33 112 202 86 179 271

4.82 34 113 203 86 180 272

4.84 34 113 204 87 181 274

4.86 34 113 205 87 181 275

4.88 34 115 205 88 181 276

4.9 34 114 206 88 183 277

4.92 33 114 207 88 183 278

4.94 33 114 208 88 183 279

4.96 33 115 210 88 184 280

4.98 33 116 211 88 185 282

5 33 115 212 88 186 283

5.02 33 115 214 88 187 284

5.04 32 115 216 88 188 286

5.06 32 116 214 88 188 288

5.08 32 117 217 88 189 288

5.1 32 117 217 88 190 291

5.12 32 119 217 88 190 291

5.14 32 119 222 88 192 291

5.16 32 120 224 88 193 296

5.18 31 121 226 88 194 298

5.2 31 122 227 88 195 300

5.22 31 122 229 88 196 303

5.24 31 122 228 88 196 306

5.26 31 123 230 88 196 306

5.28 31 123 230 88 197 308

5.3 31 124 231 88 198 309

5.32 31 125 232 88 199 310

5.34 31 126 233 88 200 312

5.36 31 126 234 88 201 313

5.38 31 125 234 88 201 315

5.4 31 126 237 88 201 316

5.42 31 127 237 88 202 320

5.44 31 129 237 88 203 321

5.46 31 129 236 88 205 322

5.48 31 130 232 88 205 323

5.5 31 130 232 88 206 325

5.52 31 131 234 88 206 325

5.54 31 130 234 88 207 327

5.56 31 129 233 88 207 328

5.58 31 131 230 88 207 328

5.6 31 133 230 88 209 328

5.62 31 133 229 88 211 329

5.64 31 134 232 88 211 329

5.66 31 132 233 88 212 332

5.68 31 134 233 88 212 333

5.7 31 134 233 88 215 333

5.72 32 134 234 88 215 333

5.74 32 134 234 89 215 334

5.76 32 134 235 89 215 336

5.78 32 133 236 89 215 338

5.8 32 132 237 89 215 339

5.82 32 133 238 89 215 341

5.84 32 133 240 89 217 342

5.86 32 131 240 89 217 344

5.88 32 131 240 89 217 346

5.9 31 130 242 89 217 347

5.92 31 128 243 89 217 349

5.94 30 127 243 89 217 350

5.96 30 131 243 89 217 351

5.98 29 134 250 89 221 351

6 29 132 250 89 224 359

6.02 29 132 251 89 224 360

6.04 29 132 253 89 224 361

6.06 28 133 255 89 224 363

6.08 28 133 255 89 225 366

6.1 28 132 256 89 226 367

6.12 28 132 257 89 226 369

6.14 27 133 259 89 226 370

6.16 26 133 259 89 227 373

6.18 25 134 259 89 227 374

6.2 26 134 260 89 229 374

6.22 25 133 258 90 229 375

6.24 24 133 258 90 229 375

6.26 24 132 259 90 229 376

6.28 24 133 262 90 229 377

6.3 23 134 262 90 230 380

6.32 23 135 263 90 231 381

6.34 23 136 265 90 232 382

6.36 23 136 266 90 233 385

6.38 21 134 267 90 233 386

6.4 21 136 272 90 233 387

6.42 20 137 271 90 236 392

6.44 20 138 276 90 237 393

6.46 20 139 278 91 238 398

6.48 19 140 281 91 239 400

6.5 19 141 281 91 240 404

6.52 19 141 283 91 241 405

6.54 19 141 283 91 241 408

6.56 18 141 284 91 242 410

6.58 18 142 285 91 242 412

6.6 18 143 285 91 244 414

6.62 18 145 284 91 245 415

6.64 18 145 284 91 247 416

6.66 18 145 283 91 247 417

6.68 18 143 283 91 247 417

6.7 18 146 285 91 247 418

6.72 18 147 285 91 250 420

6.74 18 147 284 91 251 421

6.76 18 146 286 91 252 421

6.78 18 147 286 91 252 424

6.8 18 147 286 91 253 426

6.82 18 148 287 91 254 427

6.84 18 148 286 91 255 428

6.86 18 148 288 91 256 428

6.88 18 149 288 91 257 430

6.9 18 149 288 91 258 432

6.92 18 149 287 91 258 432

6.94 18 149 288 91 260 432

6.96 18 148 290 91 260 433

6.98 18 149 289 91 260 436

7 18 150 289 91 262 436

7.02 18 150 291 91 263 437

7.04 18 150 291 91 263 440

7.06 18 149 289 91 263 441

7.08 18 149 289 91 263 443

7.1 18 146 289 91 264 444

7.12 18 148 290 91 264 445

7.14 18 150 294 91 266 447

7.16 18 152 297 91 268 451

7.18 18 151 301 91 271 454

7.2 18 150 301 91 272 459

7.22 18 149 302 91 272 461

7.24 18 149 301 91 272 462

7.26 18 149 302 91 272 463

7.28 18 149 301 91 272 465

7.3 18 150 301 91 272 465

7.32 18 152 302 91 273 465

7.34 17 153 305 91 275 467

7.36 17 155 309 91 276 471

7.38 17 154 309 92 278 476

7.4 17 154 308 92 278 477

7.42 17 154 308 92 278 477

7.44 17 155 312 92 278 477

7.46 17 154 312 92 279 482

7.48 17 153 312 92 279 483

7.5 17 152 315 92 279 484

7.52 17 150 315 92 280 488

7.54 17 151 313 92 280 489

7.56 17 152 315 92 281 489

7.58 17 151 316 92 282 491

7.6 17 152 317 92 282 492

7.62 17 150 317 92 283 493

7.64 17 150 316 92 283 494

7.66 17 149 321 92 283 495

7.68 18 149 322 92 283 500

7.7 18 151 323 93 283 501

7.72 18 152 325 93 285 502

7.74 17 152 328 93 286 504

7.76 17 151 327 93 286 508

7.78 17 151 329 93 286 508

7.8 17 151 331 93 286 511

7.82 17 152 326 93 286 514

7.84 17 153 327 93 287 514

7.86 17 152 326 93 288 515

7.88 17 153 326 93 288 515

7.9 17 152 330 93 290 517

7.92 17 152 330 93 290 521

7.94 17 153 333 93 290 522

7.96 17 152 332 93 291 525

7.98 17 154 333 93 291 525

8 17 151 333 93 293 526

8.02 17 152 333 93 293 528

8.04 16 154 333 93 294 529

8.06 14 156 335 93 296 529

8.08 15 157 335 93 298 533

8.1 15 157 335 94 299 535

8.12 15 156 337 94 299 536

8.14 15 155 335 94 299 539

8.16 16 156 337 94 301 539

8.18 16 156 339 95 303 541

8.2 16 157 340 95 303 543

8.22 16 156 337 95 304 546

8.24 14 156 336 95 304 546

8.26 14 157 334 95 304 547

8.28 14 157 337 95 305 547

8.3 14 157 339 95 306 550

8.32 14 156 339 95 307 553

8.34 14 155 337 95 307 553

8.36 14 156 337 95 307 553

8.38 14 158 338 95 308 553

8.4 14 158 335 95 310 555

8.42 14 157 337 95 310 556

8.44 14 156 338 95 310 558

8.46 14 156 341 95 312 559

8.48 14 156 340 95 313 563

8.5 14 156 340 95 313 563

8.52 14 156 339 95 314 564

8.54 14 158 338 95 315 564

8.56 14 157 338 95 317 565

8.58 14 158 336 95 317 565

8.6 15 159 337 95 319 566

8.62 15 159 336 96 320 568

8.64 15 159 335 96 321 569

8.66 15 162 336 96 321 569

8.68 15 161 336 96 324 570

8.7 15 161 336 96 324 571

8.72 15 162 337 96 324 573

8.74 15 162 339 96 325 575

8.76 15 160 339 96 326 577

8.78 15 160 337 96 326 581

8.8 15 157 338 96 326 582

8.82 14 156 338 96 326 583

8.84 14 156 336 96 326 584

8.86 14 157 336 96 327 585

8.88 14 157 337 96 329 585

8.9 14 157 338 96 329 587

8.92 14 158 338 96 330 589

8.94 15 159 337 96 331 590

8.96 15 157 336 97 332 592

8.98 15 157 335 97 334 592

9 15 157 338 97 334 594

9.02 14 156 337 97 334 597

9.04 14 157 339 97 334 598

9.06 14 156 342 97 335 601

9.08 14 159 340 97 335 605

9.1 14 160 340 97 338 607

9.12 15 160 339 97 340 608

9.14 15 163 338 98 341 608

9.16 14 163 338 98 344 609

9.18 13 163 337 98 345 609

9.2 13 163 338 98 345 609

9.22 12 164 337 98 345 611

9.24 12 167 333 98 346 612

9.26 12 167 332 98 349 613

9.28 12 167 333 98 350 613

9.3 12 168 332 98 351 615

9.32 12 167 334 98 352 615

9.34 12 166 332 98 352 617

9.36 12 167 330 98 352 618

9.38 12 166 330 98 353 618

9.4 11 166 330 98 353 618

9.42 11 166 328 98 354 619

9.44 11 165 328 98 354 620

9.46 11 164 325 98 354 622

9.48 11 164 325 98 355 622

9.5 11 167 326 98 357 623

9.52 11 166 327 98 360 626

9.54 11 166 329 98 360 628

9.56 11 166 327 98 361 630

9.58 11 167 327 98 362 632

9.6 10 167 326 98 363 632

9.62 10 167 326 98 363 632

9.64 10 168 325 98 363 633

9.66 11 167 325 98 365 634

9.68 11 167 326 99 365 636

9.7 11 169 327 99 365 637

9.72 11 168 329 99 367 640

9.74 10 167 330 99 368 643

9.76 10 167 329 99 369 644

9.78 10 167 329 99 370 644

9.8 10 167 329 99 371 645

9.82 11 166 330 99 371 647

9.84 11 165 329 100 371 648

9.86 11 165 327 100 371 649

9.88 11 166 327 100 371 649

9.9 11 167 327 100 372 650

9.92 11 167 326 100 374 653

9.94 11 167 324 100 374 654

9.96 11 167 324 100 374 654

9.98 11 167 326 100 374 655

10 11 168 326 100 374 658

10.02 11 169 327 100 375 659

10.04 11 169 327 100 377 661

10.06 11 171 324 100 377 661

10.08 11 171 322 100 379 661

10.1 11 169 320 100 380 662

10.12 11 170 319 100 382 662

10.14 11 171 319 100 383 663

10.16 11 172 320 100 384 664

10.18 11 171 320 100 385 665

10.2 11 171 318 100 386 666

10.22 11 170 318 100 386 666

10.24 10 171 320 100 387 667

10.26 10 171 322 100 388 670

10.28 10 173 320 100 388 672

10.3 10 173 320 100 390 673

10.32 10 171 319 100 390 673

10.34 10 170 320 100 392 673

10.36 10 171 318 100 392 674

10.38 10 172 319 100 394 674

10.4 10 173 318 100 395 675

10.42 10 172 318 100 398 675

10.44 10 171 318 100 398 675

10.46 10 171 318 100 398 675

10.48 10 172 318 100 398 677

10.5 10 168 317 100 399 678

10.52 10 170 310 100 399 678

10.54 10 170 308 100 402 679

10.56 10 172 304 100 402 681

10.58 10 172 306 100 404 682

10.6 10 171 304 100 404 685

10.62 10 173 303 100 404 685

10.64 10 172 306 100 406 685

10.66 10 171 304 100 407 688

10.68 10 172 305 100 407 688

10.7 10 174 306 100 410 689

10.72 10 176 308 100 412 691

10.74 10 175 305 100 414 695

10.76 10 175 305 100 415 697

10.78 10 174 305 100 416 699

10.8 10 174 306 100 416 699

10.82 10 172 308 100 416 701

10.84 10 172 308 100 416 703

10.86 10 174 307 100 416 703

10.88 10 173 306 100 418 703

10.9 10 175 306 100 418 705

10.92 10 175 301 100 420 706

10.94 10 174 302 100 420 706

10.96 10 173 300 100 420 708

10.98 10 176 299 100 420 708

11 10 175 299 100 423 708

11.02 10 173 300 100 423 708

11.04 10 173 301 100 423 709

11.06 10 173 302 100 423 711

11.08 10 173 297 100 423 713

11.1 10 174 296 100 424 714

11.12 10 175 297 100 425 716

11.14 10 174 296 100 428 717

11.16 10 176 297 100 428 717

11.18 10 176 297 100 431 718

11.2 10 175 294 100 432 718

11.22 10 178 296 100 432 718

11.24 10 179 295 100 435 720

11.26 9 181 295 100 436 722

11.28 9 180 293 100 438 722

11.3 9 178 291 100 438 722

11.32 9 178 290 100 438 723

11.34 9 180 288 100 440 724

11.36 9 179 287 100 442 724

11.38 9 178 287 100 442 726

11.4 9 176 286 100 442 727

11.42 9 173 284 100 442 728

11.44 9 171 281 100 442 729

11.46 9 172 278 100 442 729

11.48 8 171 275 100 443 729

11.5 8 171 273 100 443 729

11.52 8 171 273 100 443 730

11.54 8 172 272 100 443 731

11.56 8 172 269 100 444 731

11.58 8 172 268 100 444 732

11.6 8 172 269 100 444 733

11.62 8 172 267 100 444 735

11.64 8 172 267 100 444 736

11.66 8 173 268 100 444 737

11.68 8 173 267 100 446 739

11.7 8 172 267 100 446 739

11.72 8 173 265 100 447 739

11.74 8 173 263 100 448 739

11.76 8 173 262 100 448 739

11.78 8 172 260 100 448 739

11.8 7 171 261 100 448 739

11.82 7 171 261 100 448 740

11.84 7 171 258 100 448 741

11.86 7 172 255 100 448 742

11.88 7 173 253 100 450 742

11.9 7 173 247 100 451 742

11.92 7 173 246 100 452 742

11.94 7 173 247 100 452 743

11.96 7 174 247 100 452 744

11.98 7 175 246 100 454 744

12 7 176 246 100 455 744

12.02 7 176 244 100 456 744

12.04 7 176 245 100 456 744

12.06 7 176 243 100 456 745

12.08 7 177 243 100 456 746

12.1 7 179 245 100 457 746

12.12 6 179 243 100 459 748

12.14 6 178 243 100 459 749

12.16 6 179 242 100 460 750

12.18 6 179 242 100 461 750

12.2 6 179 242 100 462 750

12.22 6 179 241 100 463 751

12.24 6 177 240 100 464 751

12.26 5 177 240 100 465 751

12.28 5 177 238 100 465 751

12.3 5 177 239 100 465 751

12.32 5 176 239 100 465 752

12.34 5 176 240 100 465 754

12.36 5 176 239 100 466 756

12.38 5 175 242 100 467 756

12.4 5 176 241 100 467 759

12.42 5 177 242 100 468 759

12.44 5 176 244 100 469 761

12.46 5 175 240 100 469 763

12.48 5 174 241 100 469 763

12.5 5 174 242 100 469 764

12.52 5 174 240 100 470 766

12.54 6 174 239 100 473 766

12.56 6 174 238 101 475 767

12.58 6 173 236 101 475 767

12.6 7 173 236 101 475 767

12.62 7 172 234 102 475 768

12.64 6 172 231 102 476 769

12.66 6 173 231 102 477 769

12.68 6 174 230 102 478 770

12.7 6 174 226 102 479 772

12.72 6 174 224 102 480 772

12.74 6 173 225 102 480 772

12.76 6 174 224 102 480 773

12.78 6 175 224 102 482 774

12.8 6 175 224 102 483 774

12.82 6 174 221 102 484 775

12.84 6 174 220 102 484 775

12.86 6 174 220 102 484 776

12.88 6 174 219 102 484 776

12.9 6 174 219 102 485 776

12.92 6 174 221 102 486 777

12.94 6 175 219 102 486 779

12.96 6 175 218 102 487 779

12.98 6 172 215 102 487 779

13 6 174 213 102 488 779

13.02 6 174 212 102 490 779

13.04 6 176 210 102 490 779

13.06 6 175 210 102 492 779

13.08 6 176 211 102 493 779

13.1 6 175 210 102 494 781

13.12 6 175 210 102 495 781

13.14 6 175 209 102 495 783

13.16 5 175 208 102 496 785

13.18 5 173 205 102 496 785

13.2 5 172 203 102 497 785

13.22 5 172 203 102 497 785

13.24 5 172 201 102 497 785

13.26 5 172 201 102 498 785

13.28 5 172 199 102 498 786

13.3 4 171 196 102 499 786

13.32 4 170 195 102 499 787

13.34 4 171 193 102 500 787

13.36 4 171 191 102 501 787

13.38 4 170 191 102 501 787

13.4 4 170 190 102 501 788

13.42 4 170 191 102 501 788

13.44 4 169 189 102 502 789

13.46 4 168 189 102 502 790

13.48 4 169 189 102 502 790

13.5 4 169 188 102 503 790

13.52 4 170 187 102 504 790

13.54 4 168 185 102 506 790

13.56 4 167 184 102 506 790

13.58 4 166 185 102 506 790

13.6 4 165 185 102 508 792

13.62 4 165 185 102 508 793

13.64 4 165 185 102 508 794

13.66 4 165 183 102 508 795

13.68 4 166 182 102 508 797

13.7 4 165 182 102 509 797

13.72 4 165 184 102 509 797

13.74 4 165 186 102 510 799

13.76 4 164 184 102 510 801

13.78 4 163 182 102 512 801

13.8 4 162 180 102 512 801

13.82 4 161 180 102 513 801

13.84 4 162 178 102 514 802

13.86 4 163 178 102 515 802

13.88 4 162 177 102 517 803

13.9 4 161 177 102 518 803

13.92 4 161 176 102 518 803

13.94 4 162 175 102 518 803

13.96 4 161 174 102 519 803

13.98 4 162 173 102 519 803

14 4 159 171 102 520 803

14.02 4 160 172 102 520 803

14.04 3 162 168 102 521 804

14.06 3 162 169 102 524 804

14.08 3 162 167 102 525 805

14.1 3 163 168 102 526 805

14.12 3 161 165 102 527 808

14.14 3 161 165 102 527 808

14.16 3 162 163 102 527 808

14.18 3 163 162 102 528 808

14.2 3 161 162 102 529 808

14.22 3 161 162 102 529 808

14.24 3 161 162 102 529 809

14.26 3 159 160 102 529 810

14.28 3 160 159 102 530 810

14.3 3 161 158 102 532 810

14.32 3 161 156 102 533 810

14.34 3 159 155 102 534 810

14.36 3 159 155 102 535 811

14.38 3 158 155 102 535 811

14.4 3 156 154 102 535 812

14.42 3 156 154 102 535 812

14.44 3 155 154 102 536 813

14.46 3 153 153 102 536 814

14.48 3 153 154 102 536 814

14.5 3 152 154 102 536 815

14.52 3 152 153 102 536 815

14.54 2 151 152 102 536 815

14.56 2 151 150 102 536 815

14.58 2 150 149 102 536 815

14.6 2 150 149 102 536 816

14.62 2 148 150 102 536 816

14.64 2 149 150 102 537 817

14.66 2 150 149 102 538 817

14.68 2 151 149 102 540 817

14.7 2 151 149 102 542 818

14.72 2 151 149 102 543 818

14.74 2 149 150 102 543 819

14.76 2 148 149 102 543 821

14.78 2 148 149 102 544 822

14.8 2 149 148 102 544 823

14.82 2 148 149 102 545 823

14.84 2 150 146 102 545 824

14.86 2 149 144 102 547 824

14.88 2 148 142 102 547 824

14.9 2 147 142 102 548 824

14.92 2 146 140 102 548 824

14.94 2 144 141 102 548 826

14.96 2 143 141 102 548 827

14.98 2 141 140 102 548 827

15 2 140 141 102 548 827

15.02 2 140 141 102 548 828

15.04 2 140 141 102 548 828

15.06 2 140 139 102 548 828

15.08 2 140 138 102 548 828

15.1 3 139 139 102 548 828

15.12 3 139 141 103 548 829

15.14 3 137 141 103 548 831

15.16 3 137 140 103 548 831

15.18 3 137 140 103 548 831

15.2 3 137 140 103 550 831

15.22 3 136 139 103 550 831

15.24 3 133 140 103 551 832

15.26 3 132 138 103 551 833

15.28 3 131 137 103 551 833

15.3 3 131 136 103 552 833

15.32 3 133 136 103 553 833

15.34 3 132 134 103 555 833

15.36 3 131 134 103 555 834

15.38 3 132 133 103 555 834

15.4 3 133 132 103 556 835

15.42 3 133 130 103 557 835

15.44 3 132 129 103 557 835

15.46 3 132 127 103 557 835

15.48 3 132 128 103 557 835

15.5 3 132 128 103 558 836

15.52 3 133 127 103 558 837

15.54 3 132 127 103 559 837

15.56 3 130 125 103 559 837

15.58 3 128 123 103 559 837

15.6 3 128 122 103 559 838

15.62 3 128 122 103 559 838

15.64 3 128 120 103 559 839

15.66 3 127 119 103 559 839

15.68 3 127 116 103 559 839

15.7 3 127 115 103 559 839

15.72 3 127 115 103 560 839

15.74 3 124 113 103 562 839

15.76 3 122 112 103 562 839

15.78 3 122 113 103 562 839

15.8 3 122 113 103 562 840

15.82 3 122 110 103 562 840

15.84 3 122 109 103 562 841

15.86 3 121 109 103 562 841

15.88 3 121 109 103 562 841

15.9 3 122 109 103 563 841

15.92 3 122 109 103 564 841

15.94 3 122 107 103 565 841

15.96 3 122 107 103 565 841

15.98 3 122 106 103 565 843

16 3 121 106 103 566 843

16.02 3 120 106 103 566 843

16.04 3 120 106 103 566 843

16.06 3 119 106 103 566 843

16.08 3 119 104 103 566 843

16.1 3 119 104 103 566 843

16.12 3 119 103 103 566 844

16.14 3 119 102 103 566 844

16.16 3 119 102 103 566 844

16.18 3 119 103 103 567 844

16.2 3 119 102 103 567 845

16.22 3 117 102 103 567 845

16.24 3 118 101 103 567 845

16.26 3 118 100 103 568 845

16.28 3 118 100 103 568 845

16.3 3 116 99 103 568 845

16.32 3 116 98 103 569 845

16.34 3 115 98 103 569 845

16.36 3 117 97 103 569 845

16.38 3 117 97 103 572 845

16.4 3 117 97 103 572 845

16.42 3 118 96 103 572 845

16.44 3 117 95 103 573 845

16.46 3 117 95 103 573 845

16.48 3 117 93 103 574 845

16.5 3 118 91 103 574 845

16.52 4 118 91 103 575 845

16.54 4 118 87 104 575 845

16.56 4 118 87 104 576 845

16.58 4 118 86 104 576 845

16.6 4 117 86 104 576 845

16.62 4 118 86 104 576 845

16.64 5 117 86 104 577 845

16.66 5 116 86 105 577 845

16.68 5 115 87 105 577 845

16.7 4 115 87 105 577 846

16.72 4 113 88 105 577 846

16.74 4 113 88 105 577 847

16.76 4 114 87 105 577 847

16.78 4 114 85 105 578 847

16.8 4 114 85 105 578 848

16.82 4 114 85 105 579 848

16.84 4 115 85 105 579 848

16.86 4 113 84 105 580 848

16.88 4 113 84 105 580 848

16.9 4 112 82 105 581 848

16.92 4 113 82 105 581 848

16.94 4 113 81 105 582 848

16.96 4 113 81 105 583 849

16.98 4 111 79 105 583 849

17 4 111 79 105 583 849

17.02 4 112 80 105 583 849

17.04 3 109 79 105 584 850

17.06 3 108 79 105 584 850

17.08 3 107 77 105 585 850

17.1 3 107 76 105 585 850

17.12 3 107 76 105 585 850

17.14 3 107 76 105 585 850

17.16 3 104 74 105 585 850

17.18 3 102 74 105 585 850

17.2 3 100 74 105 585 850

17.22 3 100 74 105 585 850

17.24 3 98 73 105 585 850

17.26 3 98 73 105 585 850

17.28 3 96 73 105 585 850

17.3 3 97 73 105 585 850

17.32 3 98 72 105 586 850

17.34 3 100 72 105 587 850

17.36 3 101 72 105 589 850

17.38 3 102 71 105 591 850

17.4 3 102 71 105 592 850

17.42 4 101 71 105 593 850

17.44 4 102 71 106 593 850

17.46 4 101 71 106 595 851

17.48 4 100 71 106 595 851

17.5 4 101 71 106 595 851

17.52 4 100 71 106 596 851

17.54 4 101 71 106 596 851

17.56 4 103 71 106 597 851

17.58 4 103 70 106 599 851

17.6 4 102 70 106 599 851

17.62 4 102 69 106 599 851

17.64 4 101 69 106 599 851

17.66 4 102 69 106 599 851

17.68 4 103 68 106 601 851

17.7 4 103 68 106 602 851

17.72 4 103 68 106 602 852

17.74 4 104 67 106 602 852

17.76 4 104 68 106 603 852

17.78 4 104 68 106 603 853

17.8 4 104 68 106 603 853

17.82 4 105 68 106 604 853

17.84 4 103 67 106 605 853

17.86 5 103 66 106 605 853

17.88 5 102 66 107 605 853

17.9 5 102 64 107 605 853

17.92 5 101 64 107 606 853

17.94 5 101 63 107 606 853

17.96 5 101 63 107 606 853

17.98 5 102 61 107 606 853

18 5 101 61 107 607 853

18.02 5 102 61 107 607 853

18.04 5 104 60 107 608 853

18.06 5 103 60 107 610 853

18.08 5 101 59 107 610 853

18.1 5 101 59 107 611 853

18.12 5 99 59 107 611 853

18.14 5 99 58 107 611 853

18.16 5 99 58 107 611 853

18.18 5 98 58 107 611 853

18.2 5 98 58 107 611 853

18.22 5 98 57 107 611 853

18.24 5 98 57 107 611 853

18.26 5 99 55 107 611 853

18.28 5 99 54 107 612 853

18.3 5 98 54 107 612 853

18.32 5 97 53 107 612 853

18.34 5 98 53 107 612 853

18.36 5 98 52 107 614 853

18.38 5 98 52 107 614 853

18.4 5 97 52 107 614 853

18.42 5 94 51 107 614 853

18.44 5 94 51 107 614 853

18.46 5 92 51 107 614 853

18.48 5 92 49 107 614 853

18.5 5 92 49 107 614 853

18.52 5 92 49 107 614 854

18.54 5 92 49 107 614 854

18.56 5 91 49 107 614 854

18.58 5 90 49 107 614 854

18.6 5 90 50 107 614 854

18.62 5 90 49 107 614 855

18.64 5 89 49 107 614 855

18.66 5 90 47 107 614 855

18.68 5 90 45 107 615 855

18.7 5 90 45 107 615 855

18.72 5 90 45 107 615 855

18.74 5 89 45 107 615 855

18.76 5 87 45 107 616 855

18.78 5 87 44 107 616 855

18.8 5 86 42 107 616 855

18.82 5 85 41 107 616 855

18.84 5 84 41 107 617 855

18.86 6 83 41 107 619 855

18.88 6 83 41 108 619 855

18.9 6 83 41 108 620 855

18.92 6 83 41 108 620 855

18.94 6 81 39 108 621 855

18.96 6 81 39 108 621 855

18.98 6 81 38 108 621 855

19 6 81 38 108 621 855

19.02 6 81 38 108 621 855

19.04 6 81 37 108 621 855

19.06 6 81 37 108 621 855

19.08 6 82 36 108 621 855

19.1 6 83 36 108 622 855

19.12 6 84 36 108 623 855

19.14 6 84 36 108 624 855

19.16 6 83 36 108 624 855

19.18 6 82 36 108 624 855

19.2 6 82 35 108 624 855

19.22 5 82 35 108 624 855

19.24 6 81 35 108 624 855

19.26 6 81 35 109 624 855

19.28 6 80 35 109 624 855

19.3 6 81 35 109 624 855

19.32 6 80 34 109 625 855

19.34 6 80 33 109 626 855

19.36 6 80 32 109 626 855

19.38 6 80 31 109 627 855

19.4 6 80 30 109 627 855

19.42 6 80 30 109 627 855

19.44 6 80 29 109 627 855

19.46 6 80 29 109 627 855

19.48 6 80 27 109 627 855

19.5 6 82 27 109 627 855

19.52 6 81 27 109 629 855

19.54 6 81 27 109 629 855

19.56 6 81 27 109 629 855

19.58 6 80 27 109 629 855

19.6 6 80 27 109 629 855

19.62 6 80 27 109 629 855

19.64 6 81 27 109 629 855

19.66 6 81 27 109 630 855

19.68 6 81 25 109 631 855

19.7 6 79 24 109 631 855

19.72 6 79 24 109 631 855

19.74 6 79 24 109 631 855

19.76 6 79 24 109 631 855

19.78 6 80 23 109 631 855

19.8 6 80 23 109 632 855

19.82 6 80 22 109 632 855

19.84 6 81 22 109 632 855

19.86 6 81 22 109 633 855

19.88 6 81 22 109 633 855

19.9 6 82 21 109 633 855

19.92 6 82 21 109 634 855

19.94 6 82 20 109 634 855

19.96 6 82 20 109 634 855

19.98 6 81 19 109 634 855

20 6 81 19 109 634 855

20.02 6 81 18 109 634 855

20.04 6 82 17 109 634 855

20.06 6 82 17 109 635 855

20.08 6 81 17 109 635 855

20.1 6 79 17 109 635 855

20.12 6 78 18 109 635 855

20.14 6 77 18 109 635 856

20.16 6 76 17 109 635 856

20.18 6 76 17 109 635 856

20.2 6 77 17 109 635 856

20.22 6 77 16 109 636 856

20.24 6 77 16 109 636 856

20.26 6 77 16 109 636 856

20.28 6 79 16 109 636 856

20.3 6 77 16 109 638 856

20.32 6 77 15 109 638 856

20.34 6 76 15 109 638 856

20.36 6 76 15 109 638 856

20.38 6 76 15 109 638 856

20.4 6 76 15 109 638 856

20.42 6 76 15 109 639 856

20.44 6 74 14 109 639 856

20.46 6 74 14 109 639 856

20.48 6 74 14 109 640 856

20.5 6 73 14 109 640 856

20.52 6 74 14 109 640 856

20.54 6 74 14 109 641 856

20.56 6 73 14 109 641 856

20.58 6 73 14 109 641 856

20.6 6 73 14 109 641 856

20.62 6 71 14 109 641 856

20.64 6 70 14 109 641 856

20.66 6 69 14 109 641 856

20.68 6 69 14 109 641 856

20.7 6 69 14 109 641 856

20.72 5 69 14 109 641 856

20.74 4 69 14 109 641 856

20.76 4 69 14 109 641 856

20.78 4 69 14 109 641 856

20.8 4 69 14 109 641 856

20.82 4 69 14 109 641 856

20.84 4 67 14 109 641 856

20.86 4 68 14 109 641 856

20.88 4 69 14 109 642 856

20.9 5 68 14 109 643 856

20.92 5 68 13 110 643 856

20.94 5 68 13 110 643 856

20.96 5 69 12 110 643 856

20.98 5 70 12 110 644 856

21 5 70 11 110 645 856

21.02 5 71 10 110 645 856

21.04 5 71 10 110 646 856

21.06 5 72 10 110 646 856

21.08 5 72 10 110 647 856

21.1 5 72 10 110 647 856

21.12 5 72 10 110 647 856

21.14 5 71 10 110 647 856

21.16 5 70 10 110 647 856

21.18 6 69 10 110 647 856

21.2 6 70 9 111 647 856

21.22 6 70 8 111 648 856

21.24 6 71 8 111 648 856

21.26 6 71 8 111 649 856

21.28 6 72 8 111 649 856

21.3 6 71 8 111 650 856

21.32 6 70 8 111 650 856

21.34 6 69 8 111 650 856

21.36 6 68 8 111 650 856

21.38 6 69 8 111 650 856

21.4 6 69 7 111 651 856

21.42 6 68 7 111 651 856

21.44 6 68 7 111 651 856

21.46 6 68 7 111 651 856

21.48 6 66 7 111 651 856

21.5 6 65 7 111 651 856

21.52 6 65 7 111 651 856

21.54 6 65 7 111 651 856

21.56 6 64 7 111 651 856

21.58 6 64 7 111 651 856

21.6 5 65 7 111 651 856

21.62 5 64 7 111 652 856

21.64 5 64 7 111 652 856

21.66 5 64 7 111 652 856

21.68 5 63 7 111 652 856

21.7 5 61 7 111 652 856

21.72 5 59 7 111 652 856

21.74 5 58 6 111 652 856

21.76 5 59 6 111 652 856

21.78 5 59 6 111 653 856

21.8 5 59 5 111 653 856

21.82 5 59 5 111 653 856

21.84 5 60 5 111 653 856

21.86 5 60 5 111 654 856

21.88 5 60 5 111 654 856

21.9 5 60 5 111 654 856

21.92 5 59 5 111 654 856

21.94 5 58 5 111 654 856

21.96 5 57 5 111 654 856

21.98 5 56 5 111 654 856

22 5 55 5 111 654 856

22.02 5 55 5 111 654 856

22.04 5 56 5 111 654 856

22.06 5 56 5 111 655 856

22.08 5 56 5 111 655 856

22.1 5 55 5 111 655 856

22.12 5 55 5 111 655 856

22.14 5 55 5 111 655 856

22.16 5 54 5 111 655 856

22.18 5 55 5 111 655 856

22.2 5 54 5 111 656 856

22.22 5 54 5 111 656 856

22.24 5 53 5 111 656 856

22.26 5 52 5 111 656 856

22.28 5 52 5 111 656 856

22.3 5 52 5 111 656 856

22.32 5 52 5 111 657 856

22.34 5 53 4 111 657 856

22.36 5 54 4 111 658 856

22.38 5 54 4 111 659 856

22.4 5 54 4 111 659 856

22.42 5 54 4 111 659 856

22.44 5 53 4 111 659 856

22.46 5 53 4 111 659 856

22.48 5 53 4 111 659 856

22.5 5 52 4 111 659 856

22.52 5 52 4 111 659 856

22.54 5 52 4 111 659 856

22.56 5 52 4 111 659 856

22.58 5 52 4 111 659 856

22.6 5 51 4 111 659 856

22.62 5 51 4 111 659 856

22.64 5 52 4 111 659 856

22.66 5 51 4 111 660 856

22.68 5 51 4 111 660 856

22.7 5 51 4 111 660 856

22.72 5 50 3 111 660 856

22.74 5 50 3 111 660 856

22.76 5 49 3 111 660 856

22.78 5 49 3 111 660 856

22.8 5 50 1 111 660 856

22.82 5 50 1 111 661 856

22.84 6 50 1 111 661 856

22.86 6 50 1 112 661 856

22.88 5 50 1 112 661 856

22.9 4 48 1 112 661 856

22.92 4 49 1 112 661 856

22.94 4 49 1 112 662 856

22.96 4 49 1 112 662 856

22.98 4 49 1 112 662 856

23 4 48 1 112 662 856

23.02 4 48 1 112 662 856

23.04 4 48 1 112 662 856

23.06 4 49 1 112 662 856

23.08 4 48 1 112 663 856

23.1 4 48 1 112 663 856

23.12 4 48 1 112 663 856

23.14 4 48 1 112 663 856

23.16 4 46 1 112 663 856

23.18 4 46 1 112 663 856

23.2 4 46 1 112 663 856

23.22 4 45 1 112 663 856

23.24 4 45 1 112 663 856

23.26 3 45 1 112 663 856

23.28 3 45 1 112 663 856

23.3 3 45 1 112 663 856

23.32 3 45 1 112 663 856

23.34 3 45 1 112 663 856

23.36 3 44 1 112 663 856

23.38 3 44 1 112 663 856

23.4 3 42 1 112 663 856

23.42 3 42 1 112 663 856

23.44 3 42 1 112 664 856

23.46 3 41 1 112 664 856

23.48 3 41 1 112 664 856

23.5 3 41 1 112 664 856

23.52 3 41 1 112 664 856

23.54 3 42 1 112 664 856

23.56 3 42 1 112 665 856

23.58 3 43 1 112 665 856

23.6 3 42 1 112 666 856

23.62 3 42 1 112 666 856

23.64 3 42 1 112 667 856

23.66 3 41 1 112 667 856

23.68 3 41 1 112 667 856

23.7 3 40 1 112 667 856

23.72 3 40 1 112 667 856

23.74 3 39 1 112 667 856

23.76 3 39 1 112 667 856

23.78 3 38 1 112 667 856

23.8 3 38 1 112 667 856

23.82 3 38 1 112 667 856

23.84 3 38 1 112 667 856

23.86 3 38 1 112 667 856

23.88 3 38 1 112 667 856

23.9 3 38 1 112 667 856

23.92 3 38 1 112 667 856

23.94 3 38 1 112 667 856

23.96 3 37 1 112 667 856

23.98 3 37 1 112 667 856

24 3 37 1 112 667 856

24.02 3 37 1 112 667 856

24.04 3 36 1 112 667 856

24.06 3 35 1 112 667 856

24.08 3 35 1 112 667 856

24.1 3 35 1 112 667 856

24.12 3 35 1 112 667 856

24.14 3 36 1 112 668 856

24.16 3 36 1 112 669 856

24.18 3 36 1 112 669 856

24.2 3 36 1 112 669 856

24.22 3 36 1 112 669 856

24.24 3 36 1 112 669 856

24.26 3 36 1 112 669 856

24.28 3 36 1 112 669 856

24.3 3 36 1 112 669 856

24.32 3 36 1 112 669 856

24.34 3 36 1 112 669 856

24.36 3 36 1 112 669 856

24.38 3 35 1 112 669 856

24.4 3 34 1 112 669 856

24.42 3 34 1 112 669 856

24.44 3 34 1 112 669 856

24.46 3 34 1 112 669 856

24.48 3 34 1 112 669 856

24.5 3 34 1 112 669 856

24.52 3 33 1 112 669 856

24.54 3 33 1 112 669 856

24.56 3 33 1 112 669 856

24.58 3 33 1 112 669 856

24.6 3 33 1 112 669 856

24.62 3 33 1 112 669 856

24.64 3 33 1 112 669 856

24.66 3 33 1 112 669 856

24.68 3 33 1 112 669 856

24.7 3 32 1 112 669 856

24.72 3 31 1 112 669 856

24.74 3 31 1 112 669 856

24.76 3 31 1 112 669 856

24.78 3 31 1 112 669 856

24.8 3 31 1 112 669 856

24.82 3 31 0 112 669 856

24.84 3 31 0 112 669 856

24.86 3 31 0 112 669 856

24.88 3 31 0 112 669 856

24.9 3 31 0 112 669 856

24.92 3 30 0 112 669 856

24.94 3 29 0 112 669 856

24.96 3 29 0 112 669 856

24.98 3 29 0 112 669 856

25 3 29 0 112 669 856

25.02 3 29 0 112 669 856

25.04 3 29 0 112 669 856

25.06 2 28 0 112 669 856

25.08 2 26 0 112 669 856

25.1 2 26 0 112 669 856

25.12 2 25 0 112 669 856

25.14 2 25 0 112 669 856

25.16 2 25 0 112 669 856

25.18 2 25 0 112 669 856

25.2 2 25 0 112 669 856

25.22 2 24 0 112 669 856

25.24 2 23 0 112 669 856

25.26 2 23 0 112 669 856

25.28 2 23 0 112 669 856

25.3 2 23 0 112 669 856

25.32 1 23 0 112 669 856

25.34 1 23 0 112 669 856

25.36 1 21 0 112 669 856

25.38 1 22 0 112 669 856

25.4 1 22 0 112 670 856

25.42 1 22 0 112 670 856

25.44 1 22 0 112 670 856

25.46 1 22 0 112 670 856

25.48 1 22 0 112 670 856

25.5 1 22 0 112 670 856

25.52 1 22 0 112 670 856

25.54 1 22 0 112 670 856

25.56 1 23 0 112 670 856

25.58 1 23 0 112 671 856

25.6 1 23 0 112 671 856

25.62 1 23 0 112 671 856

25.64 1 23 0 112 671 856

25.66 1 23 0 112 671 856

25.68 1 23 0 112 671 856

25.7 1 23 0 112 671 856

25.72 1 23 0 112 671 856

25.74 1 23 0 112 671 856

25.76 1 23 0 112 671 856

25.78 1 23 0 112 671 856

25.8 1 23 0 112 671 856

25.82 1 22 0 112 671 856

25.84 1 22 0 112 671 856

25.86 1 22 0 112 671 856

25.88 1 22 0 112 671 856

25.9 1 22 0 112 671 856

25.92 1 22 0 112 671 856

25.94 1 22 0 112 671 856

25.96 1 22 0 112 671 856

25.98 1 22 0 112 671 856

26 1 22 0 112 671 856

26.02 1 22 0 112 671 856

26.04 1 22 0 112 671 856

26.06 1 22 0 112 671 856

26.08 1 22 0 112 671 856

26.1 1 22 0 112 671 856

26.12 1 22 0 112 671 856

26.14 1 22 0 112 671 856

26.16 1 22 0 112 671 856

26.18 1 21 0 112 671 856

26.2 1 21 0 112 671 856

26.22 1 21 0 112 671 856

26.24 1 21 0 112 671 856

26.26 1 20 0 112 671 856

26.28 1 20 0 112 671 856

26.3 1 19 0 112 671 856

26.32 1 19 0 112 671 856

26.34 1 19 0 112 671 856

26.36 1 18 0 112 671 856

26.38 1 16 0 112 671 856

26.4 1 16 0 112 671 856

26.42 1 16 0 112 671 856

26.44 1 17 0 112 671 856

26.46 1 16 0 112 672 856

26.48 1 16 0 112 672 856

26.5 1 16 0 112 672 856

26.52 1 16 0 112 672 856

26.54 1 16 0 112 672 856

26.56 1 16 0 112 672 856

26.58 1 16 0 112 672 856

26.6 1 15 0 112 672 856

26.62 1 15 0 112 672 856

26.64 1 15 0 112 672 856

26.66 1 15 0 112 672 856

26.68 1 15 0 112 672 856

26.7 1 15 0 112 672 856

26.72 1 16 0 112 672 856

26.74 1 16 0 112 673 856

26.76 1 16 0 112 673 856

26.78 1 16 0 112 673 856

26.8 1 16 0 112 673 856

26.82 1 16 0 112 673 856

26.84 1 16 0 112 673 856

26.86 1 16 0 112 673 856

26.88 1 16 0 112 673 856

26.9 0 16 0 112 673 856

26.92 0 16 0 112 673 856

26.94 0 16 0 112 673 856

26.96 0 16 0 112 673 856

26.98 0 15 0 112 673 856

27 0 15 0 112 673 856

27.02 0 15 0 112 673 856

27.04 0 15 0 112 673 856

27.06 0 15 0 112 673 856

27.08 0 15 0 112 673 856

27.1 0 15 0 112 673 856

27.12 0 15 0 112 673 856

27.14 0 15 0 112 673 856

27.16 0 15 0 112 673 856

27.18 0 15 0 112 673 856

27.2 0 14 0 112 673 856

27.22 0 14 0 112 673 856

27.24 0 13 0 112 673 856

27.26 0 13 0 112 673 856

27.28 0 12 0 112 673 856

27.3 0 12 0 112 673 856

27.32 0 12 0 112 673 856

27.34 0 11 0 112 673 856

27.36 0 11 0 112 673 856

27.38 0 11 0 112 673 856

27.4 0 11 0 112 673 856

27.42 0 11 0 112 673 856

27.44 0 11 0 112 673 856

27.46 0 11 0 112 673 856

27.48 0 11 0 112 673 856

27.5 0 11 0 112 673 856

27.52 0 11 0 112 673 856

27.54 0 11 0 112 673 856

27.56 0 11 0 112 673 856

27.58 0 11 0 112 673 856

27.6 0 11 0 112 673 856

27.62 0 10 0 112 673 856

27.64 0 10 0 112 673 856

27.66 0 10 0 112 673 856

27.68 0 9 0 112 673 856

27.7 0 9 0 112 673 856

27.72 0 9 0 112 673 856

27.74 0 9 0 112 673 856

27.76 0 9 0 112 673 856

27.78 0 9 0 112 673 856

27.8 0 9 0 112 673 856

27.82 0 9 0 112 673 856

27.84 0 9 0 112 673 856

27.86 0 9 0 112 673 856

27.88 0 9 0 112 673 856

27.9 0 9 0 112 673 856

27.92 0 9 0 112 673 856

27.94 0 9 0 112 673 856

27.96 0 9 0 112 673 856

27.98 0 9 0 112 673 856

28 0 9 0 112 673 856

28.02 0 9 0 112 673 856

28.04 0 9 0 112 673 856

28.06 0 9 0 112 673 856

28.08 0 9 0 112 673 856

28.1 0 8 0 112 673 856

28.12 0 8 0 112 673 856

28.14 0 8 0 112 673 856

28.16 0 8 0 112 673 856

28.18 0 8 0 112 673 856

28.2 0 8 0 112 673 856

28.22 0 8 0 112 673 856

28.24 0 8 0 112 673 856

28.26 0 8 0 112 673 856

28.28 0 8 0 112 674 856

28.3 0 8 0 112 674 856

28.32 0 8 0 112 674 856

28.34 0 8 0 112 674 856

28.36 0 8 0 112 674 856

28.38 0 8 0 112 674 856

28.4 0 8 0 112 674 856

28.42 0 8 0 112 674 856

28.44 0 7 0 112 674 856

28.46 0 7 0 112 674 856

28.48 0 7 0 112 674 856

28.5 0 7 0 112 674 856

28.52 0 7 0 112 674 856

28.54 0 7 0 112 674 856

28.56 0 6 0 112 674 856

28.58 0 6 0 112 674 856

28.6 0 6 0 112 674 856

28.62 0 6 0 112 674 856

28.64 0 5 0 112 674 856

28.66 0 5 0 112 674 856

28.68 0 5 0 112 674 856

28.7 0 5 0 112 674 856

28.72 0 5 0 112 674 856

28.74 0 5 0 112 674 856

28.76 0 5 0 112 674 856

28.78 0 5 0 112 674 856

28.8 0 5 0 112 674 856

28.82 0 5 0 112 674 856

28.84 0 5 0 112 674 856

28.86 0 5 0 112 674 856

28.88 0 5 0 112 674 856

28.9 0 5 0 112 674 856

28.92 0 5 0 112 674 856

28.94 0 5 0 112 674 856

28.96 0 5 0 112 674 856

28.98 0 5 0 112 674 856

29 0 5 0 112 674 856

29.02 0 5 0 112 674 856

29.04 0 5 0 112 674 856

29.06 0 5 0 112 674 856

29.08 0 5 0 112 674 856

29.1 0 5 0 112 674 856

29.12 0 5 0 112 674 856

29.14 0 5 0 112 674 856

29.16 0 5 0 112 674 856

29.18 0 5 0 112 674 856

29.2 0 5 0 112 674 856

29.22 0 5 0 112 674 856

29.24 0 5 0 112 674 856

29.26 0 5 0 112 674 856

29.28 0 5 0 112 674 856

29.3 0 5 0 112 674 856

29.32 0 5 0 112 674 856

29.34 0 5 0 112 674 856

29.36 0 5 0 112 674 856

29.38 0 5 0 112 674 856

29.4 0 5 0 112 674 856

29.42 0 5 0 112 674 856

29.44 0 5 0 112 674 856

29.46 0 5 0 112 674 856

29.48 0 5 0 112 674 856

29.5 0 5 0 112 674 856

29.52 0 5 0 112 674 856

29.54 0 5 0 112 674 856

29.56 0 5 0 112 674 856

29.58 0 5 0 112 674 856

29.6 0 5 0 112 674 856

29.62 0 5 0 112 674 856

29.64 0 5 0 112 674 856

29.66 0 5 0 112 674 856

29.68 0 5 0 112 674 856

29.7 0 5 0 112 674 856

29.72 0 5 0 112 674 856

29.74 0 5 0 112 674 856

29.76 0 5 0 112 674 856

29.78 0 5 0 112 674 856

29.8 0 5 0 112 674 856

29.82 0 5 0 112 674 856

29.84 0 5 0 112 674 856

29.86 0 3 0 112 674 856

29.88 0 3 0 112 674 856

29.9 0 3 0 112 674 856

29.92 0 3 0 112 674 856

29.94 0 3 0 112 674 856

29.96 0 3 0 112 674 856

29.98 0 3 0 112 674 856

30 0 3 0 112 674 856
