# Supplementary material for: Sustainability of religious communities
Source: PLoS One. 2021 May 7;16(5):e0250718. doi: 10.1371/journal.pone.0250718 (PMC8104927; doi:10.1371/journal.pone.0250718)
Supplement: S5 Fig — (DOCX) [file pone.0250718.s005.docx]

Time Birth Death Church Mem.

1995 15.7 5.3 2.1033E6

1996 15 5.2 2.1457E6

1997 14.4 5.2 2.18821E6

1998 13.6 5.2 2.20797E6

1999 13 5.2 2.24533E6

2000 13.3 5.2 2.28311E6

2001 11.6 5 2.32841E6

2002 10.2 5.1 2.329E6

2003 10.2 5.1 2.39535E6

2004 9.8 5 2.48972E6

2005 8.9 5 2.53943E6

2006 9.2 5 2.64885E6

2007 10 5 2.68681E6

2008 9.4 5 2.69942E6

2009 9 5 2.80258E6

2010 9.4 5.1 2.85231E6

2011 9.4 5.1 2.85213E6

2012 9.6 5.3 2.81053E6

2013 8.6 5.3 2.80891E6

2014 8.6 5.3 2.81057E6

2015 8.6 5.4 2.7891E6

2016 7.9 5.5 2.7309E6

2017 7 5.5 2.6277E6

2018 6.4 5.8 2.55423E6
