# Supplementary material for: Sustainability of religious communities
Source: PLoS One. 2021 May 7;16(5):e0250718. doi: 10.1371/journal.pone.0250718 (PMC8104927; doi:10.1371/journal.pone.0250718)
Supplement: S6 Fig — (PPTX) [file pone.0250718.s006.pptx]

## Slide 1
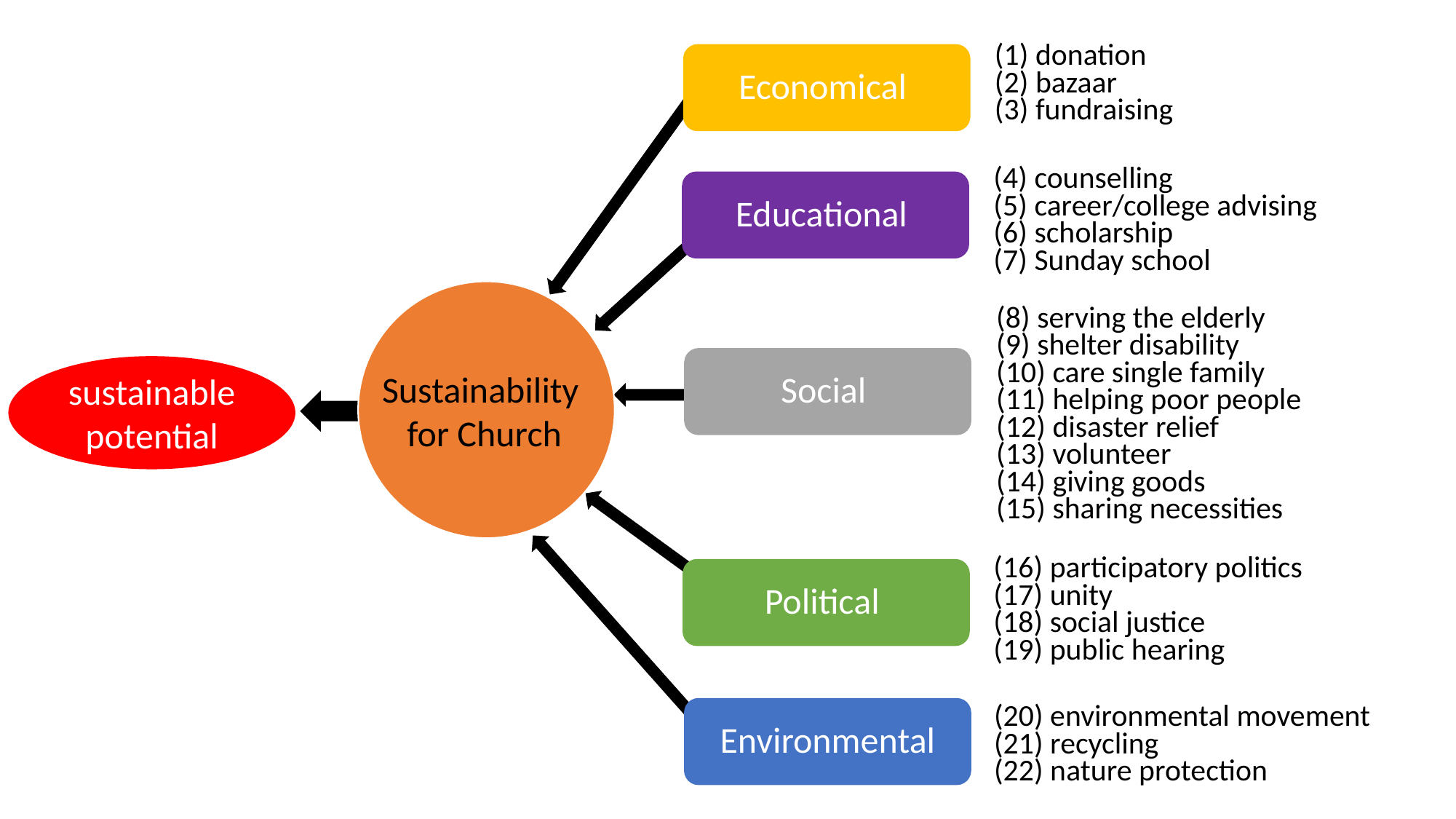

(1) donation
(2) bazaar
(3) fundraising
Economical
(4) counselling
(5) career/college advising
(6) scholarship
(7) Sunday school
Educational
(8) serving the elderly
(9) shelter disability
(10) care single family
(11) helping poor people
(12) disaster relief
(13) volunteer
(14) giving goods
(15) sharing necessities
Social
sustainable
potential
Sustainability
for Church
(16) participatory politics
(17) unity
(18) social justice
(19) public hearing
Political
Environmental
(20) environmental movement
(21) recycling
(22) nature protection
