# Supplementary material for: Sustainability of religious communities
Source: PLoS One. 2021 May 7;16(5):e0250718. doi: 10.1371/journal.pone.0250718 (PMC8104927; doi:10.1371/journal.pone.0250718)
Supplement: S7 Fig — (DOCX) [file pone.0250718.s007.docx]

Time SP

1995 0.91

1996 0.94

1997 0.95

1998 0.91

1999 0.92

2000 1.07

2001 0.95

2002 1

2003 1.03

2004 1.04

2005 1

2006 1.04

2007 1.03

2008 1.17

2009 1.16

2010 1.26

2011 1.24

2012 1.14

2013 1.26

2014 1.14

2015 1.16

2016 1.16

2017 1.11

2018 1.16
