# Supplementary material for: Sustainability of religious communities
Source: PLoS One. 2021 May 7;16(5):e0250718. doi: 10.1371/journal.pone.0250718 (PMC8104927; doi:10.1371/journal.pone.0250718)
Supplement: S8 Fig — (DOCX) [file pone.0250718.s008.docx]

Time PCK CMs

1995 2.1033E6 2.103E6

1996 2.1457E6 2.19978E6

1997 2.18821E6 2.29172E6

1998 2.20797E6 2.37385E6

1999 2.24533E6 2.42978E6

2000 2.28311E6 2.47721E6

2001 2.32841E6 2.56309E6

2002 2.329E6 2.59897E6

2003 2.39535E6 2.6375E6

2004 2.48972E6 2.67538E6

2005 2.53943E6 2.70575E6

2006 2.64885E6 2.71463E6

2007 2.68681E6 2.72629E6

2008 2.69942E6 2.72831E6

2009 2.80258E6 2.75961E6

2010 2.85231E6 2.77808E6

2011 2.85213E6 2.81613E6

2012 2.81053E6 2.84022E6

2013 2.80891E6 2.82524E6

2014 2.81057E6 2.82934E6

2015 2.7891E6 2.79403E6

2016 2.7309E6 2.75346E6

2017 2.6277E6 2.70123E6

2018 2.55423E6 2.62609E6

2019 -- 2.54967E6

2020 -- 2.48304E6

2021 -- 2.42524E6

2022 -- 2.37539E6

2023 -- 2.33272E6

2024 2.2965E6

2025 2.26606E6

2026 2.24075E6

2027 2.21997E6

2028 2.20315E6

2029 2.18972E6

2030 2.17915E6
