# Supplementary material for: Sustainability of religious communities
Source: PLoS One. 2021 May 7;16(5):e0250718. doi: 10.1371/journal.pone.0250718 (PMC8104927; doi:10.1371/journal.pone.0250718)
Supplement: S9 Fig — (DOCX) [file pone.0250718.s009.docx]

Time CMPCK CMs1.3f0.1 CMs1.3f0.05 CMs1.2f0.1 CMs1.2f0.05 CMBase

1995 2.1033E6 2.103E6 2.103E6 2.103E6 2.103E6 2.103E6

1996 2.1457E6 2.19591E6 2.19591E6 2.19591E6 2.19591E6 2.19591E6

1997 2.18821E6 2.28189E6 2.28189E6 2.28189E6 2.28189E6 2.28189E6

1998 2.20797E6 2.3614E6 2.3614E6 2.3614E6 2.3614E6 2.3614E6

1999 2.24533E6 2.43402E6 2.43402E6 2.43402E6 2.43402E6 2.43402E6

2000 2.28311E6 2.50049E6 2.50049E6 2.50049E6 2.50049E6 2.50049E6

2001 2.32841E6 2.56142E6 2.56142E6 2.56142E6 2.56142E6 2.56142E6

2002 2.329E6 2.61469E6 2.61469E6 2.61469E6 2.61469E6 2.61469E6

2003 2.39535E6 2.65922E6 2.65922E6 2.65922E6 2.65922E6 2.65922E6

2004 2.48972E6 2.69757E6 2.69757E6 2.69757E6 2.69757E6 2.69757E6

2005 2.53943E6 2.72921E6 2.72921E6 2.72921E6 2.72921E6 2.72921E6

2006 2.64885E6 2.75537E6 2.75537E6 2.75537E6 2.75537E6 2.75537E6

2007 2.68681E6 2.77612E6 2.77612E6 2.77612E6 2.77612E6 2.77612E6

2008 2.69942E6 2.79332E6 2.79332E6 2.79332E6 2.79332E6 2.79332E6

2009 2.80258E6 2.804E6 2.804E6 2.804E6 2.804E6 2.804E6

2010 2.85231E6 2.80878E6 2.80878E6 2.80878E6 2.80878E6 2.80878E6

2011 2.85213E6 2.80971E6 2.80971E6 2.80971E6 2.80971E6 2.80971E6

2012 2.81053E6 2.80652E6 2.80652E6 2.80652E6 2.80652E6 2.80652E6

2013 2.80891E6 2.79723E6 2.79723E6 2.79723E6 2.79723E6 2.79723E6

2014 2.81057E6 2.78267E6 2.78267E6 2.78267E6 2.78267E6 2.78267E6

2015 2.7891E6 2.76478E6 2.76478E6 2.76478E6 2.76478E6 2.76478E6

2016 2.7309E6 2.74287E6 2.74287E6 2.74287E6 2.74287E6 2.74287E6

2017 2.6277E6 2.7161E6 2.7161E6 2.7161E6 2.7161E6 2.7161E6

2018 2.55423E6 2.68431E6 2.68431E6 2.68431E6 2.68431E6 2.68431E6

2019 -- 2.6763E6 2.68561E6 2.68766E6 2.69775E6 2.64737E6

2020 -- 2.66498E6 2.68339E6 2.6872E6 2.70713E6 2.60862E6

2021 -- 2.65065E6 2.67794E6 2.68315E6 2.71269E6 2.56837E6

2022 -- 2.63359E6 2.66951E6 2.67577E6 2.71464E6 2.52692E6

2023 -- 2.61406E6 2.65838E6 2.6653E6 2.71323E6 2.48452E6

2024 2.59234E6 2.64481E6 2.65199E6 2.70872E6 2.44142E6

2025 2.56868E6 2.62906E6 2.63611E6 2.70135E6 2.39784E6

2026 2.54333E6 2.61137E6 2.61789E6 2.69138E6 2.35397E6

2027 2.5165E6 2.59196E6 2.59757E6 2.67903E6 2.30998E6

2028 2.48842E6 2.57105E6 2.5754E6 2.66456E6 2.26603E6

2029 2.45927E6 2.54883E6 2.55159E6 2.64819E6 2.22225E6

2030 2.42923E6 2.5255E6 2.52634E6 2.63012E6 2.17875E6
