# Supplementary material for: Sustainability of religious communities
Source: PLoS One. 2021 May 7;16(5):e0250718. doi: 10.1371/journal.pone.0250718 (PMC8104927; doi:10.1371/journal.pone.0250718)
Supplement: S1 Table — (DOCX) [file pone.0250718.s010.docx]

| Time | Economical | | | Educational | | | | Social | | | | | | | | | Political | | | | | Environmental | | | Total articles | |
| --- | --- | --- | --- | --- | --- | --- | --- | --- | --- | --- | --- | --- | --- | --- | --- | --- | --- | --- | --- | --- | --- | --- | --- | --- | --- | --- |
| Year | (1) donation | (2) bazaar | (3) fundraising | (4) counselling | (5) career/college advising | (6) scholarship | (7) Sunday school | (8) serving the elderly | (9) shelter disability | (10) care single family | (11) helping poor people | (12) disaster relief | (13) volunteer | (14) giving goods | (15) sharing necessities | (16) participatory politics | | (17) unity | (18) social justice | (19) public hearing | (20) environmental movement | | (21) recycling | (22) nature protection |  |  |
| 1995 | 36 | 9 | 98 | 21 | 24 | 64 | 13 | 53 | 5 | 2 | 50 | 103 | 20 | 6 | 13 | 65 | | 122 | 72 | 21 | 98 | | 7 | 12 | 3996 |  |
| 1996 | 28 | 12 | 134 | 27 | 31 | 60 | 12 | 64 | 6 | 1 | 87 | 88 | 44 | 7 | 27 | 59 | | 100 | 95 | 13 | 109 | | 19 | 16 | 4434 |  |
| 1997 | 25 | 18 | 173 | 33 | 27 | 71 | 10 | 55 | 1 | 5 | 88 | 66 | 51 | 9 | 13 | 61 | | 138 | 80 | 13 | 110 | | 10 | 15 | 4494 |  |
| 1998 | 25 | 26 | 131 | 27 | 26 | 60 | 16 | 55 | 7 | 6 | 94 | 86 | 34 | 6 | 19 | 35 | | 106 | 65 | 25 | 85 | | 18 | 6 | 4218 |  |
| 1999 | 31 | 33 | 132 | 33 | 26 | 87 | 29 | 79 | 9 | 7 | 87 | 92 | 91 | 20 | 10 | 58 | | 193 | 76 | 32 | 98 | | 11 | 18 | 5426 |  |
| 2000 | 66 | 33 | 161 | 47 | 53 | 128 | 29 | 103 | 12 | 17 | 107 | 116 | 118 | 22 | 30 | 81 | | 239 | 114 | 22 | 141 | | 13 | 24 | 6241 |  |
| 2001 | 47 | 34 | 150 | 40 | 43 | 105 | 33 | 107 | 18 | 10 | 158 | 106 | 141 | 20 | 37 | 55 | | 183 | 86 | 14 | 137 | | 22 | 24 | 6579 |  |
| 2002 | 64 | 52 | 220 | 68 | 45 | 117 | 36 | 177 | 23 | 18 | 163 | 196 | 143 | 42 | 51 | 99 | | 232 | 88 | 22 | 136 | | 11 | 20 | 8095 |  |
| 2003 | 77 | 48 | 230 | 53 | 61 | 149 | 38 | 127 | 25 | 22 | 142 | 197 | 187 | 38 | 47 | 101 | | 237 | 121 | 32 | 153 | | 26 | 27 | 8325 |  |
| 2004 | 121 | 55 | 257 | 77 | 48 | 145 | 26 | 139 | 29 | 30 | 132 | 194 | 231 | 36 | 43 | 151 | | 234 | 111 | 67 | 195 | | 24 | 33 | 9162 |  |
| 2005 | 132 | 50 | 211 | 54 | 41 | 130 | 15 | 115 | 25 | 29 | 129 | 194 | 188 | 24 | 68 | 62 | | 190 | 117 | 24 | 160 | | 27 | 34 | 8004 |  |
| 2006 | 137 | 62 | 216 | 65 | 43 | 148 | 10 | 131 | 21 | 19 | 124 | 201 | 180 | 41 | 67 | 99 | | 165 | 111 | 31 | 152 | | 22 | 27 | 7991 |  |
| 2007 | 172 | 49 | 238 | 55 | 48 | 162 | 18 | 133 | 30 | 29 | 137 | 199 | 267 | 38 | 90 | 124 | | 197 | 128 | 27 | 176 | | 27 | 36 | 9219 |  |
| 2008 | 243 | 79 | 265 | 47 | 67 | 222 | 66 | 157 | 18 | 52 | 170 | 255 | 398 | 36 | 146 | 163 | | 283 | 176 | 18 | 271 | | 28 | 43 | 10905 |  |
| 2009 | 301 | 86 | 275 | 54 | 77 | 252 | 26 | 157 | 29 | 74 | 215 | 209 | 428 | 56 | 184 | 131 | | 328 | 179 | 37 | 223 | | 55 | 41 | 11768 |  |
| 2010 | 313 | 76 | 358 | 65 | 87 | 267 | 33 | 126 | 40 | 81 | 227 | 366 | 428 | 58 | 212 | 142 | | 373 | 179 | 47 | 236 | | 38 | 58 | 12103 |  |
| 2011 | 390 | 71 | 280 | 58 | 80 | 283 | 42 | 127 | 44 | 83 | 188 | 324 | 365 | 62 | 200 | 163 | | 307 | 221 | 27 | 210 | | 41 | 53 | 11693 |  |
| 2012 | 458 | 75 | 185 | 58 | 77 | 258 | 42 | 122 | 45 | 82 | 188 | 214 | 307 | 46 | 198 | 196 | | 264 | 187 | 40 | 202 | | 34 | 51 | 11653 |  |
| 2013 | 368 | 51 | 306 | 66 | 76 | 258 | 53 | 137 | 49 | 122 | 195 | 296 | 315 | 56 | 210 | 141 | | 278 | 277 | 36 | 209 | | 32 | 41 | 11314 |  |
| 2014 | 415 | 97 | 306 | 84 | 82 | 245 | 45 | 149 | 43 | 138 | 154 | 318 | 410 | 81 | 226 | 166 | | 350 | 337 | 37 | 181 | | 19 | 47 | 13730 |  |
| 2015 | 499 | 74 | 319 | 87 | 115 | 315 | 61 | 126 | 23 | 91 | 134 | 339 | 351 | 61 | 201 | 173 | | 265 | 204 | 57 | 185 | | 32 | 49 | 13024 |  |
| 2016 | 359 | 67 | 196 | 72 | 82 | 206 | 49 | 132 | 27 | 85 | 131 | 236 | 314 | 65 | 215 | 117 | | 200 | 162 | 27 | 127 | | 27 | 43 | 10127 |  |
| 2017 | 290 | 41 | 157 | 61 | 76 | 186 | 33 | 77 | 33 | 99 | 114 | 235 | 315 | 90 | 183 | 160 | | 166 | 182 | 21 | 119 | | 27 | 46 | 9805 |  |
| 2018 | 291 | 55 | 192 | 53 | 82 | 225 | 55 | 89 | 34 | 132 | 110 | 250 | 265 | 74 | 229 | 119 | | 150 | 161 | 28 | 153 | | 36 | 51 | 9788 |  |
