# Supplementary material for: Sustainability of religious communities
Source: PLoS One. 2021 May 7;16(5):e0250718. doi: 10.1371/journal.pone.0250718 (PMC8104927; doi:10.1371/journal.pone.0250718)
Supplement: Attachment — Submitted filename: Response to Reviewers.docx [file pone.0250718.s012.docx]

PONE-D-20-30060R1
Sustainability of Religious Population
PLOS ONE

Dear Dr. Siew Ann Cheong,

Thank you for inviting us to submit a revised draft of our manuscript entitled, "Sustainability of Religious Population" to PLOS ONE. We also appreciate the time and effort you and each of the reviewers have dedicated to providing insightful feedback on ways to strengthen our paper. Thus, it is with great pleasure that we resubmit our article for further consideration. We have incorporated changes that reflect the detailed suggestions you have provided. We also hope that our edits and the responses we provide below satisfactorily address all the issues and concerns you and the reviewers have noted.

Again, thank you for giving us the opportunity to strengthen our manuscript with your valuable comments and queries. We have worked hard to incorporate your feedback and hope that these revisions persuade you to accept our submission.

Sincerely,

Jae-Woo Lee

**Editor suggestions:**

Please address the remaining concerns of Reviewer 2. Also, 'Riligious Population' sounds awkward in the title, and 'Religious' is misspelled. Will the authors consider 'Religious Communities' instead?

RESPONSE: The authors change the title to “Sustainability of Religious Communities.”

**Comments to the Author**

Reviewer #1: Most of the points I commented on before have been improved and the paper is now very easy to read and understand.
In particular, it is easier to understand the allocation of topics, the position of the research in the timeline, and the correspondence and relationship with previous research in Korea.

We appreciate the useful review.

Reviewer #2: I appreciate the efforts the authors put into responding to my earlier comments. The removal of text in the first few pages and addition of other text throughout does improve the framing. The focus now seems more about the public image and functional purpose of the church in Korean society, and how improving these assists with membership sustainability by making the un-churched more open to joining the church. A key phrase in helping me understand this was on page 12: “Sustainable potential s reflects how amicable unbelievers are to the Christian faith, and integrates social factors in a society.” This contrasts to previous approaches, which were focused on conversion potential via the level of enthusiasm for evangelism among current members.
Unfortunately, the contribution of the sustainability potential still doesn’t become clear until nearly half-way through the paper. There is still a problem that sustainability is introduced in a way that many readers may find confusing. The introduction should be about the sustainability of church membership through many avenues including conversion efforts, not how religious values or beliefs are related to the field of sustainability.

Response

We add a new paragraph explaining the sustainability potential in the introduction part as following:

“If the new believer's faith is not strengthened, they will lose their passion for faith after a certain period of time. New believers are more likely to lose networks with unbelieving friends, and they will have a hard time forming new networks within the church. Therefore, sustainability potential represents the degree to which unbelievers contact believers and form trust. It represents the degree of trade-off between what unbelievers lose as they gain faith and what they gain in social networks and new communities.”

The first two paragraphs should be written to highlight the way that past literature tends to emphasize the enthusiasm for evangelism as a key factor in church growth, and why church image and improving it through positive social engagement and activism is also important. I recommend the work of Mara Einstein on the religious branding of the United Methodist Church (start with article titled: “The Evolution of Religious Branding”) to assist in this framing.

Response:

Religious communities carry out a variety of methods with the aim of maintaining their own sustainability. The United Methodist Church used religious branding to enhance the public reputation and to change the position of the traditional denomination. It demonstrated the need of marketing tool for the sustainability of religious institutions (Einstein, 2011). Oosthuizen and Lategan (2015) pointed out that church leaders often had difficulty performing the basic management tasks they expected because leaders had insufficient management principles and skills as an organization. The authors argued that church and denomination leaders should participate in management principles and skills education for more effective, efficient and sustainable management. Körösvölgyi (2017) addressed that as the Christian population declined, it was necessary to look at the positive and negative experiences affecting the sustainability of the church amid changes in the world map such as global warming, urbanization, uneven distribution of population and wealth, and migration. The author argued that if the place of worship as sacred building equipped with an efficient architectural concept to be better suited to the community, it could have a positive impact on the sustainability of the church.

Einstein M. The evolution of religious branding. Social compass. 2011 Sep;58(3):331-8.

Oosthuizen AJ, Lategan LO. " Managing the household of God": The contribution from management sciences to the sustainability of the church as an organization. Stellenbosch Theological Journal. 2015;1(2):551-68.

Körösvölgyi Z. The Sustainable Church: A New Way to Look at the Place of Worship. Periodica Polytechnica Architecture. 2017;48(2):93-100.

Church planting is a good strategy if the church is to be sustainable through conversion. Churches wishing to grow should invest equally in member satisfaction and recruitment (conversion). In addition, the ministry of welcoming new believers and discipling them should be balanced (Paas, 2018). De Wetter (2011) and Roozen (2008) described the health and growth of the church as the concept of church vitality. They insisted that the spiritual vitality of worship through enthusiastic, committed or active members could be the catalyst for membership growth.

Young-Gi Chai (2010) showed through the small group ministry called as the house church in the Seoul Baptist Church of Houston that the growth and sustainability of the church could be caused by conversion of non-believers. By converting the traditional church into a small group-centered church, the church focused on converting non-believers and raising beginners as disciples. Churches participating in house church ministry reeducated pastors and lay leaders by holding seminars and conferences after establishing the institute named as The House Church Ministries with Chai. The Seoul Baptist Church of Houston also supported that other churches could sustain the small group ministry by providing short-term training.

Church enthusiasts lead to successful evangelism by adding new members when they are primarily involved in the conversion of unbelievers. It is the sustainability of the church that drives growth through its ability to reproduce itself (Hayward, 2005; Medcalfe and Sharp, 2012).

Paas S. A Case Study of Church Growth by Church Planting in Germany: Are They Connected?. International Bulletin of Mission Research. 2018 Jan;42(1):40-54.

De Wetter D, Gochman I, Luss R, Sherwood R. UMC call to action: Vital congregations research project. UMC. org. 2010 Jun 28.

Roozen DA. American congregations 2008. Hartford Institute for Religion Research, Hartford Seminary; 2007.

Chai YG, Chai D. A New Testament church in the 21st century: The house church. GLPI; 2010.

Hayward J. A general model of church growth and decline. Journal of Mathematical Sociology. 2005 Jul 1;29(3):177-207.

Medcalfe S, Sharp C. Enthusiasm and congregation growth: Evidence from the United Methodist Church. International Journal of Business and Social Science. 2012 May 1;3(9).

I disagree with the other reviewer regarding who the likely audience for the paper is. Given the complexity and novelty of SD/ABM methods, it seems unlikely to me that the audience will be church leaders and pastors. For that reason, I am not concerned with how the audience may perceive epidemiological and HIV model. I also do not see the usefulness of figure 7, it is nearly impossible to read.

Response:

We believe that the SD/ABM method contributes to scientific humanities research as well as statistical research. Since Fig. 7 is difficult to look at, we have only described the relevant content without Fig. 7.

Among my lesser concerns is that the new sections of the manuscript need a copy edit, including the typo to the submitted title.
If the authors are successful in framing the article, it may be a very well-rounded contribution.

Response:

We corrected the title typo and conducted a copy edit the modified and added parts.
